# Quantitative Genetics of CTCF Binding Reveal Local Sequence Effects and Different Modes of X-Chromosome Association

**DOI:** 10.1371/journal.pgen.1004798

**Published:** 2014-11-20

**Authors:** Zhihao Ding, Yunyun Ni, Sander W. Timmer, Bum-Kyu Lee, Anna Battenhouse, Sandra Louzada, Fengtang Yang, Ian Dunham, Gregory E. Crawford, Jason D. Lieb, Richard Durbin, Vishwanath R. Iyer, Ewan Birney

**Affiliations:** 1 The Wellcome Trust Sanger Institute, Wellcome Trust Genome Campus, Hinxton, Cambridgeshire, United Kingdom; 2 Center for Systems and Synthetic Biology, Institute for Cellular and Molecular Biology, Department of Molecular Biosciences, University of Texas at Austin, Austin, Texas, United States of America; 3 The European Molecular Biology Laboratory, The European Bioinformatics Institute (EMBL-EBI), Wellcome Trust Genome Campus, Hinxton, Cambridgeshire, United Kingdom; 4 Institute for Genome Sciences and Policy, and Department of Pediatrics, Division of Medical Genetics, Duke University, Durham, North Carolina, United States of America; 5 Department of Biology and Lineberger Comprehensive Cancer Center, The University of North Carolina at Chapel Hill, Chapel Hill, North Carolina, United States of America; Georgia Institute of Technology, United States of America

## Abstract

Associating genetic variation with quantitative measures of gene regulation offers a way to bridge the gap between genotype and complex phenotypes. In order to identify quantitative trait loci (QTLs) that influence the binding of a transcription factor in humans, we measured binding of the multifunctional transcription and chromatin factor CTCF in 51 HapMap cell lines. We identified thousands of QTLs in which genotype differences were associated with differences in CTCF binding strength, hundreds of them confirmed by directly observable allele-specific binding bias. The majority of QTLs were either within 1 kb of the CTCF binding motif, or in linkage disequilibrium with a variant within 1 kb of the motif. On the X chromosome we observed three classes of binding sites: a minority class bound only to the active copy of the X chromosome, the majority class bound to both the active and inactive X, and a small set of female-specific CTCF sites associated with two non-coding RNA genes. In sum, our data reveal extensive genetic effects on CTCF binding, both direct and indirect, and identify a diversity of patterns of CTCF binding on the X chromosome.

## Author Summary

We have systematically measured the effect of normal genetic variation present in a human population on the binding of a specific chromatin protein (CTCF) to DNA by measuring its binding in 51 human cell lines. We observed a large number of changes in protein binding that we can confidently attribute to genetic effects. The corresponding genetic changes are often clustered around the binding motif for CTCF, but only a minority are actually within the motif. Unexpectedly, we also find that at most binding sites on the X chromosome, CTCF binding occurs equally on both the X chromosomes in females at the same level as on the single X chromosome in males. This finding suggests that in general, CTCF binding is not subject to global dosage compensation, the process which equalizes gene expression levels from the two female X chromosomes and the single male X.

## Introduction

A major challenge in human genetics is to understand the mechanisms that link variation in genomic sequence to phenotypes of interest, including disease. Since 2005, a growing number of genome-wide association studies (GWAS) have associated both disease and normal phenotypes with over 9,800 single nucleotide polymorphisms (SNPs) [1]. Association studies can identify either causative variants or SNPs in linkage disequilibrium (LD) with the causative variant. Considerable effort has been invested in identifying potential causative variants, because this is essential to understanding the mechanistic route from the change in genomic sequence to final phenotype. The majority of the loci that have been found are not in strong linkage disequilibrium with a protein coding variant, suggesting that a change in a non-protein coding DNA sequence is often responsible for the phenotypic effect [2].

One route to finding intermediates between genotype and whole organism phenotype is to study the effect of genetic variants on gene regulation. New technologies such as microarrays and RNA sequencing (RNA-seq) have enabled quantification of transcript levels for every gene in a genome. Similarly, genome wide measurements of transcription factor occupancy and chromatin structure via chromatin immunoprecipitation followed by sequencing (ChIP-seq) [3] and DNase I hypersensitivity assays [4]–[7] have made it possible to quantify the state of upstream activities important for regulating transcription. Using DNase I hypersensitivity and binding assays for the CTCF transcription factor on two family trios with known genome sequences, we showed that allele-specific binding patterns consistent with strong genetic effects could be readily measured at heterozygous sites [8]. Other studies have shown allele specific binding of RNA polymerase and NF-κB binding measured across a small number of individuals [9], or of a wider range of transcription factors in a single cell line [10]. Similarly, differences between mouse strains in binding of PU-1 and CEBP/α at enhancer regions correlate with sequence differences and adjacent gene expression [11]. Intriguingly, some sites with prominent SNPs in the binding motifs of CTCF did not show a genetic effect in a study of its binding across an extended family [12]. Reciprocally, differences in transcriptor factor binding were seen between closely related species even where there was no sequence difference in the binding region [13].

In order to examine these phenomena further, and infer potential causative connections to disease GWAS results, we need to identify specific cases where a genetic variant affects a binding site. To do this we can use a genetic association study, as in GWAS, that searches for statistical association of genetic variants to quantitative measurements taken across samples. The variants with statistically significant association are known as quantitative trait loci (QTLs). When applied to transcript expression levels as the measurements on 60 or more samples, this approach has identified thousands of expression quantitative trait loci (eQTLs) [14]–[16]. A QTL study of human open chromatin [17] found 8,902 DNase I hypersensitivity sites that were correlated with genetic variants. However, there are currently no systematic association studies of how genetic variation in human populations affects the binding pattern of a specific transcription factor. Here we carry out such a study.

To identify transcription factor binding QTLs, we measured the binding of CTCF across a panel of cell lines. CTCF is a highly conserved multifunctional protein that serves as both a transcription factor as well an insulator binding protein, preventing interactions between enhancers and promoters and demarcating chromatin domains. Working with cohesin, CTCF can also mediate chromosomal looping interactions, and is involved in imprinting as well as X-inactivation (see [18], [19] for reviews). There have been extensive locus specific studies [20]–[26] and specific genome wide screens [27]–[30] demonstrating the different roles of CTCF in different circumstances. Studies by ourselves and others have shown the extent of genetic effects on CTCF binding in families [8], [12], although specific loci underlying these effects have not been identified.

We used ChIP-seq to measure CTCF binding in 51 lymphoblastoid cell lines (LCLs) from the HapMap CEU population, each of which had already been sequenced as part of the 1000 Genomes Project [2] and had been subjected to RNA-seq analysis [31]. Our data and analysis identified thousands of CTCF binding QTLs across the human genome. These data, together with the available full genome sequence of the cell lines, allowed us to explore parameters of genetic effects on protein-DNA binding. For example, we defined the relationship of the QTL location to the TF binding motif, estimated the relative impact of substitutions and insertions/deletions (indels), and measured whether allele-specific differences are indicative of population-wide variation.

Furthermore, our study revealed a previously uncharacterized mode of CTCF binding on the X chromosome. In human females (XX), one X chromosome is randomly inactivated and does not express most protein coding RNAs (reviewed in [32]). Thus for most X chromosome genes, both male and female cells have just one active locus, resulting in dosage compensation between the two sexes. The X-inactivation process requires expression of the non-coding RNA Xist from the inactive X. When we looked at CTCF binding on the X chromosome across our samples, we observed three distinct classes of CTCF binding sites. One major class was sensitive to X inactivation such that the active X showed stronger binding. Another class showed similar binding by CTCF on both X chromosomes, and the third, minor class of sites exhibited female specific binding.

## Results

### Analysis of CTCF binding in 51 genotyped individuals reveals thousands of binding QTLs

**Figure 1:**
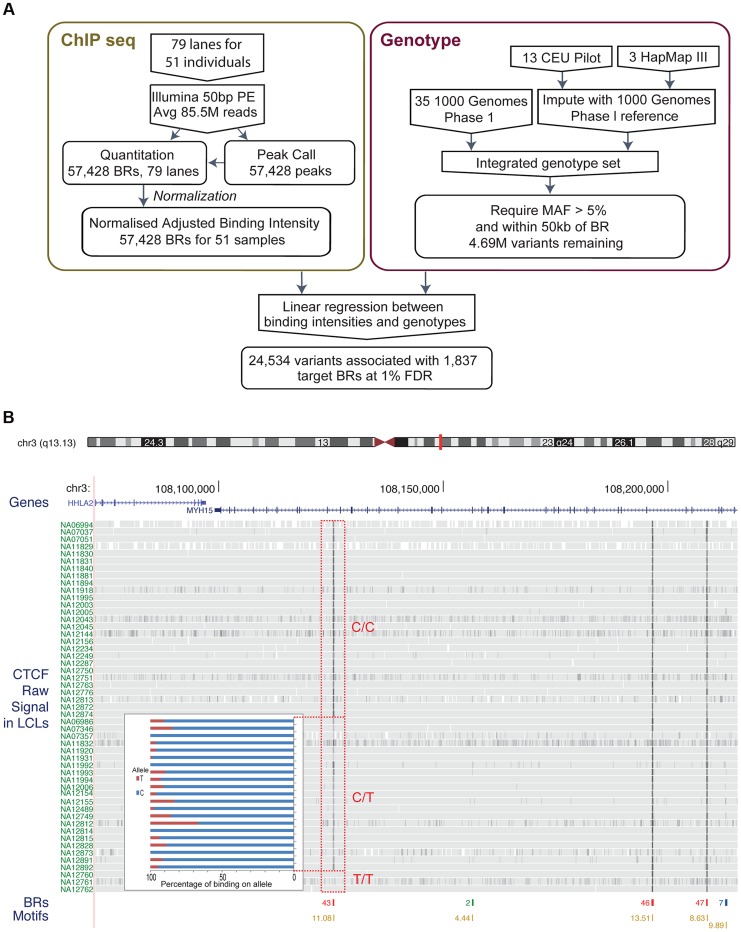
A. Flow chart indicating the overall design of the experiment. CTCF binding was identified and quantified using ChIP-seq data. Raw data was normalised to adjust for variations introduced by the experimental steps. A consolidated genotype set was produced using genotype data from three data sources. A linear regression approach was then used to identify associations between genotype dosages and CTCF binding intensities. The result set was obtained at a 1% FDR level. B. Overview of the binding intensities of a binding site across samples in three genotype groups of the associated SNP. ChIP-seq signal from the samples is aligned as tracks for this region of chromosome 3. The greyness is proportional to fragments mapped at the position, indicating binding intensity, with dark grey indicating high fragment count. Samples are grouped by their genotype at SNP rs936266, C/C, C/T or T/T, respectively. Binding sites were identified, as shown in the binding region track along with the number of samples passing the peak calling threshold. The colours of the binding regions represent the consistency of identifying the binding region across samples. Specifically, red binding regions were identified in 10 or more cell lines, blue binding regions in 5–9 cell lines and green binding regions in 2–4 cell lines. Finally the bottom track shows the corresponding CTCF motifs, with quality score attached to each site. The binding intensity decreases for T heterozygotes and further for T homozygotes. The inset panel shows allele-specific binding for the C and T allele (blue and red, respectively) in the heterozygous individuals (C/T) as percentage of the total count. Binding intensities consistently favour the C allele over the T allele.

We performed ChIP-seq on extracted chromatin from genotyped LCLs as previously described [33] except that we sequenced the DNA fragments from both ends (Figure 1) (Materials and Methods). We quantified binding to binding regions similarly to previous work [33] but pooled all the samples and identified a composite set of binding regions with detectable CTCF binding at low threshold. We then counted the sequence fragments that overlap each binding region in each individual, and normalised the signal to correct for systematic biases as in Degner *et al* [17]. We discarded binding regions that showed very little inter individual variance or had only one or two individuals with significant binding scores. Overall, our normalized data showed good consistency across all 51 individuals, as well as variation in signal sufficient to motivate QTL analysis (Figure 1B).

To measure the variance due to growth differences between the cells, we grew two individual cell lines as four independent cultures started on four consecutive days. There was higher correlation between these biological replicates from the same individual than between samples from different individuals, although all data sets were modestly correlated as expected for CTCF ChIP-seq (Figure S1). We next examined the data to see whether there were any systematic biases between samples. A principal component analysis identified some systematic variance, with a particularly strong first component (24.1%, Figure S2) that on investigation was correlated to known experimental batches. We therefore removed the first principal component, significantly improving the recovery of QTLs (Figure S3, Methods). We used the resulting normalised adjusted binding intensity (NABI) for subsequent analyses.

**Figure 2:**
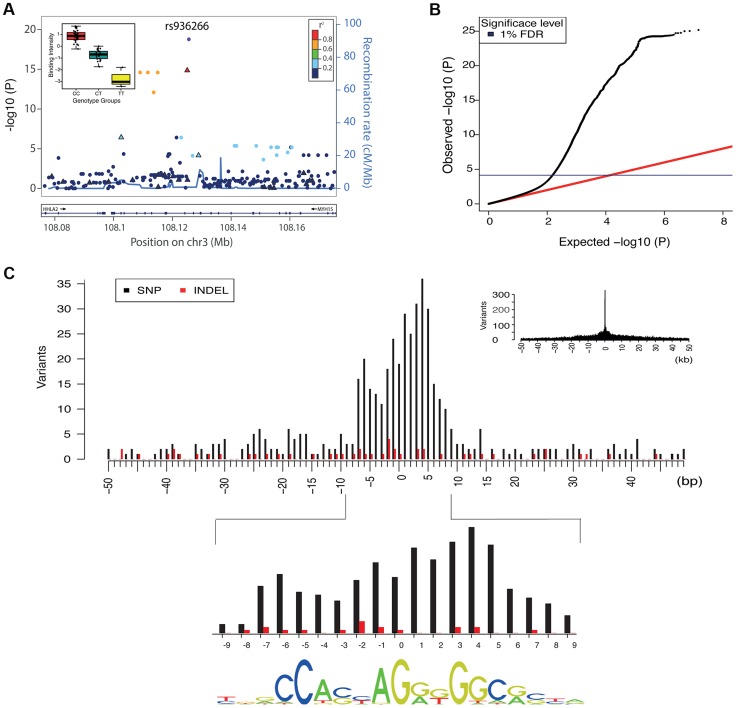
Overall properties of CTCF QTLs. A. All associations for all variants in the region of the binding region at chr3:108125397–108125829. SNPs are shown as solid circles and INDELs are shown as triangles, coloured by r^2^. Inset is boxplot showing the normalised adjusted binding intensity (NABI) for the different possible genotypes of SNP rs936266. Genotype is strongly associated with the binding intensity of the binding region at chr3:108125397–108125829 (P = 1.69E-19), with the C allele favoring binding. B. A quantile-quantile plot showing the distribution of the observed (y-axis) compared to the expected P values(x-axis). The red line is the distribution of the P values from the null model. The blue line on the y-axis shows the 1% FDR level determined by the q value method [34]. C. The density of QTL variants with respect to distance from the motif of the associated binding regions. Density plots are shown at kb (inset) and base pair resolution (main plot). SNPs and INDELs are shown as black and red bars respectively. For these cases the QTL density correlates with the information content of the motif (Spearman rank rho = 0.63) shown at the bottom.

To discover QTLs, we looked for correlations between the NABI measures and SNPs and small biallelic insertion or deletion (indel) variants within 50 kb of the relevant binding region, using a linear model (Table 1, Example in Figure 2A; see Methods). As expected, the majority of variants do not have a significant association with variation in CTCF binding, with the linear model P-value distribution following the expected distribution (>95% of tests, fraction of the overlap between the black line and red line, Figure 2B). When samples are permuted, the distribution of the test statistic falls on the expected line (see Figure S4). Using a non-parametric statistic we saw similar P values (Figure S5). Using a Bonferroni adjusted threshold of P<3.8E-9 (See details on association testing in Methods) we find 509 binding regions with significant QTLs. Using a more liberal False Discovery Rate (FDR) [34] approach to take advantage of the smaller number of effectively independent tests occurring in these limited cis-regions, we discovered 1,837 binding regions (3% of total binding regions) with at least one significant variant at the 1% FDR level; relaxing the threshold to 10% FDR we discover 6,747 binding regions (12% of the total) (Table 1).

**Table 1:** Summary statistics of the CTCF QTL scan.

**Study Parameters**	
**Traits (BRs)**	57,428
**Variants**	4,687,317
**SNPs**	4,250,881
**Indels**	436,436
**Study results**	
**BRs**	1,837
**Variants**	24,534
**SNPs**	22,954
**Indels**	1,580
**GWAS overlaps**	61
**eQTL overlaps**	366

We chose to focus further analysis on the 1% FDR threshold as this provided ample QTLs from which to derive insights. We only considered one association per binding region, because the small number of samples meant that there was insufficient power for a conditional analysis for secondary associations in almost all cases. Within this set of associations, the genetic variant accounted for a substantial fraction of the variation in CTCF binding (median R square 0.38).

We summarised the collective set of variants which might be involved in each binding region association as being the cluster of SNPs within one order of magnitude of the P-value of the lead variant. 24,534 variants were identified in at least one cluster at the 1% FDR level, 13.4 variants on average per binding region (Table 1). As expected, these variants were mainly clustered around the target binding region, and when a CTCF binding motif could be identified (1341 of the 1837 cases) and a cluster QTL variant was present in the motif, the frequency was correlated with the information content of the motif (Figure 2C), as seen previously [12]. However, only a minority of significant binding regions had a QTL candidate within the motif (433/1341), and in only a small majority of cases there was a QTL within 1 kb (747/1341), of the binding region (Table 2).

**Table 2:** CTCF QTLs with associated variants in different distance ranges.

Significance	Binding region count	Motifs in binding regions	QTL in Motif	≤1 kb	≤10 kb	≤30 kb
10% FDR	6747	5260	550	1386	2583	4057
1% FDR	1837	1341	433	747	1023	1199
BONF	509	360	164	258	322	341

We explored further the cases where there was no proximal variant in the cluster. There was not a substantial difference in genotype quality around the associated binding regions in these cases compared to binding regions with proximal effects, suggesting that there is not a large missing data problem. When considering all 1000 Genomes Project variants including those with allele frequency below 5%, in 95.5% of these cases, there was a proximal variant within 1 kb of the binding region in linkage disequilibrium (LD) with the distal lead variant, where LD was defined as the absolute value of D′>0.5. In approximately half of these cases the P-value of the proximal association either fell just outside the one order of magnitude threshold to fall in the cluster, or was just under the FDR threshold (Figure S6). In the 99 such cases where such a proximal variant was within the CTCF binding motif, the position of the variant was correlated with the information content of the position in the motif (Figure S7). Therefore a substantial fraction of the apparently distal cases appear to be explained by proximal cases. However still only a minority can be explained by variants in the binding motif.

We also conducted the analysis excluding short indels to replicate the more commonplace association analysis using only SNPs. In an indel-free analysis we would have missed QTLs in 67 binding regions entirely (∼5% of significant binding regions), and for 56 additional binding regions the closest observed explanatory SNP would have been over 1 kb away from the motif inside the peak. For these SNPs, there is usually a short indel with similar direct P-value inside the binding region. We further explored whether another cause for distal QTL effects could be due to the distal variant affecting a second neighbouring binding region, which in turn influenced the primary binding region, but there was only one case where we could find any evidence for this model (Figure S8). We additionally investigated the cases where there exist binding interactions between the QTL binding region and the neighboring region. We observed corresponding changes in histone modifications depending on the direction of the interactions between two binding regions (Figure S9, S10).

The effect size distribution with respect to allele frequency shows increased effect sizes for lower frequency SNPs, with a clear absence of large effects of common alleles (Figure S11). There is no statistical difference in effect size distribution between SNP and indel variants (Figure S11).

**Figure 3:**
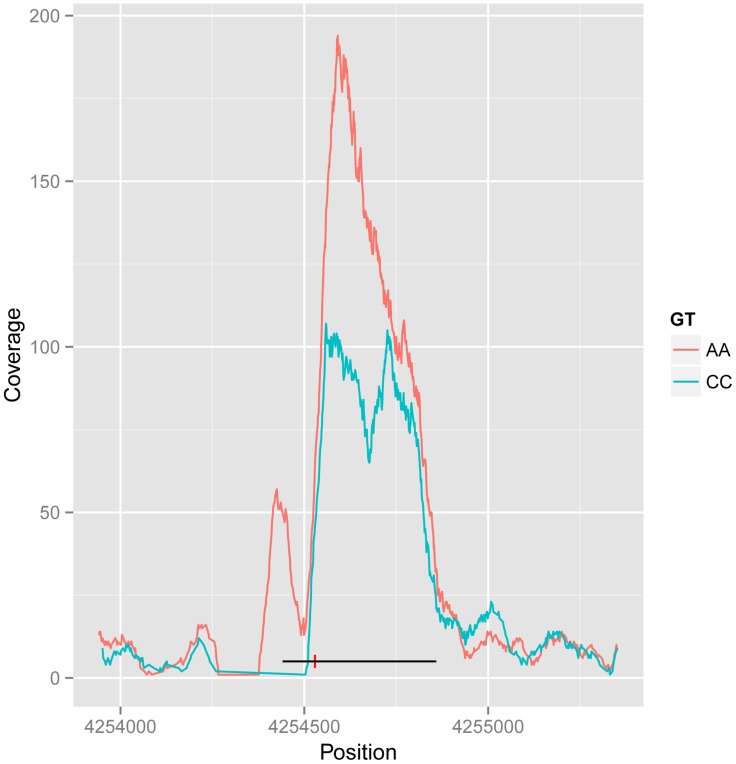
Example of CTCF peak shape QTL. Reads for samples in each homozygous genotype group at QTL rs11935835 were merged (AA and CC, respectively), and the average CC genotype profile is plotted in blue and the average AA genotype in orange. The AA genotype has stronger overall binding, with a second peak to the left, whereas the CC genotype has a double peak. The heterozygote has intermediate profile between these two (not visualized in this figure). The binding region is marked as a black line with the SNP position marked by a red vertical dash.

The dual-end sequencing of the ChIP-seq fragments provides the resolution to discover specific binding modes that influence the spatial distribution of the recovered fragments. To analyse this, we characterised ChIP-seq binding regions by metrics that summarised the extent of the peak and the position of the summit on a per individual basis, and used these additional metrics as phenotypes in a quantitative trait analysis using the methods described above. We found 25 shifts in peak shape driven by a genetic locus at the 1% FDR. Ten cases were also associated with a change in peak height. An example is shown in Figure 3, with the two homozygous genotypes showing the creation of a new associated peak, and merging of a double peak, and from visual inspection the other cases also look as if they can be explained as two CTCF peaks in close proximity, one or both of which is under *cis*-genetic control.

There are 61 CTCF QTL variants that overlap with disease and trait associated variants from other studies (GWAS Catalog [1]). In particular there is a disproportionate overlap with immune system related diseases (20 variants; Chi-sq P-value 1.7E-9). This is consistent with the lymphocyte origin of LCLs, and suggests a causal pathway for CTCF binding in the molecular aetiology of the disease phenotype in at least some cases. However many of these variants fall within the MHC locus, and a full causal analysis would need to take account of the complex LD structure there.

In summary, these results are consistent with previous studies [9], [10], [12], [13] that observed substantial variation in transcription factor binding within and between species, only a minority of which could be accounted for by genetic differences in the binding site. We also found that only 25.7% of our QTLs could be explained by a genetic variant in the motif. The majority of the remainder can be explained by changes within 1 kb of the motif, consistent with observations that transcription factor binding differences between mouse strains are more likely if there are genetic differences within 200 bp of the binding site [11]. However there remain some genetic associations for which we are not able to identify any proximal candidate, suggesting that longer range influences can make some contribution to CTCF binding.

### Allele-specific bias analysis of CTCF binding provides independent confirmation of QTLs

This data set represents an excellent resource to directly examine allele-specific biases in TF binding at heterozygous sites in a larger set of individuals than previous studies [8]. Allele-specific binding refers to statistically significant biases in binding to the two alleles in a diploid cell, at sites where a heterozygous polymorphism allows the two alleles to be distinguished. Allele-specific binding thus is an independent way of assessing how genetic variants at binding sites might affect binding variation. Although the two alleles at heterozygous SNPs are normally referred to as the reference or alternate allele (referring to which base is found in the reference genome sequence and which is the alternate base), here we chose to categorize the two alleles as ancestral (shared with chimp) or derived (human specific). This has two advantages. First, any residual effect of biases in aligning sequence reads to the reference allele will be minimized. Second, measuring allele-specific binding in terms of the ancestral and derived allele provides information about how evolutionary changes might affect CTCF binding.

**Figure 4:**
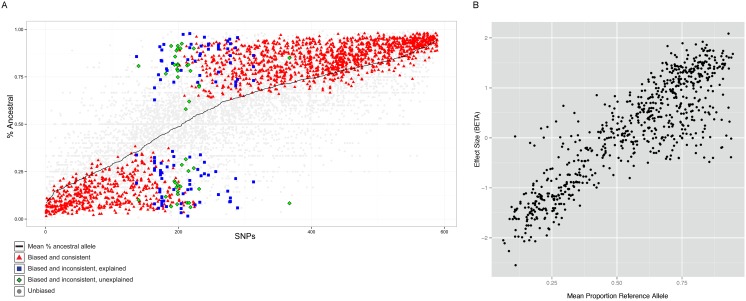
Properties of allele specific CTCF sites. A. Summary of allele-specific analysis. SNP loci that show significant allele-specific CTCF binding in at least 2 samples are included. The y-axis represents the proportion of the total read counts from the ancestral allele. The 589 SNP loci are ordered by mean proportion ancestral allele for all heterozygous samples (black line). Heterozygous samples that do not pass the allele-specificity threshold are shown as light gray points. Significant and consistent allele-specific samples (ie. the binding bias is toward the same allele) are represented by red triangle points. Significant but inconsistent samples are either blue square (inconsistency explained by the nature of the site) or green diamond (inconsistency unexplained). B. Allele-specificity correlates with QTL effect size (BETA). The mean proportion reference allele count for all heterozygous samples at SNP loci that show significant allele-specificity in at least 2 samples are plotted against the QTL effect size (BETA) at that locus. Only the BETA values from associations where the SNP is located within the associated binding region are shown.

After processing the reads, we identified allele-specific statistically sites using a binomial null model of equal occupancy of both alleles at heterozygous sites, using a 5% FDR corrected threshold (see Methods). This process identified 589 SNPs that have replicated in at least two individuals showing significant allele-specific bias. We examined the allele counts of all heterozygous individuals at these 589 SNPs. For most sites (91.5%) the allele-specific biases were consistent between individuals, confirming the predominantly genetic basis of allele-specific binding (Figure 4A). At such sites, the same ancestral or derived allele was preferred for binding across 2 or more individuals.

However, there were 50 (8.5%) sites which showed significant but opposite allele-specific biases between two or more individuals. Six of these 50 sites could potentially be explained by virtue of being close to loci known to be subject to allelic exclusion (the Immunoglobulin heavy chain), a process that affects one allele randomly (see Discussion). One site lies in the KCNQ1 imprinted locus, where the regulatory status depends on parent of origin rather than genotype. The 46 other sites at which the allele-specific binding bias switches between individuals (Table S1) could represent new random allelic exclusion loci or imprinted sites, or could arise because the site at which we see allele specificity is incompletely linked with the causal variant [35]. We tested whether there was a SNP which specifically explained the allele specific switching site; for 28 cases this was the case. We are not able to directly test whether any of these sites could be due to imprinting because parent-of-origin information is not available for the heterozygous alleles of these individuals.

Interestingly, a significant majority (68%, P<1E-16) of the SNPs showed increased binding to the ancestral allele (Figure 4A). Alignment bias towards the reference allele has been reported before [8] and because the ancestral allele is more likely to be the reference allele, the increased binding to the ancestral allele could be the result of the alignment bias. To rule out this possibility, we analyzed the cases where the ancestral allele is the alternate allele and found that the binding bias remained towards the ancestral allele (Figure S12). Additionally, we repeated the allele-specific analysis after using a variant-aware aligner (see Methods). The results were largely identical to what we observed as described above, indicating that the preference for the ancestral allele is not a trivial outcome of any alignment bias (Figure S13).

The allele-specific signal at binding regions (intra-individual measurements) mostly correlated linearly with the QTL effect size (inter-individual measurements) (Figure 4B). There were however exceptions to this, and these were mainly cases in which there was an allele-specific signal but not inter-individual QTL. We did not observe QTLs with strong effect size in binding regions that did not show strong allele-specificity (Figure S14).

### Interactions between CTCF and the X chromosome suggest novel binding modes

**Figure 5:**
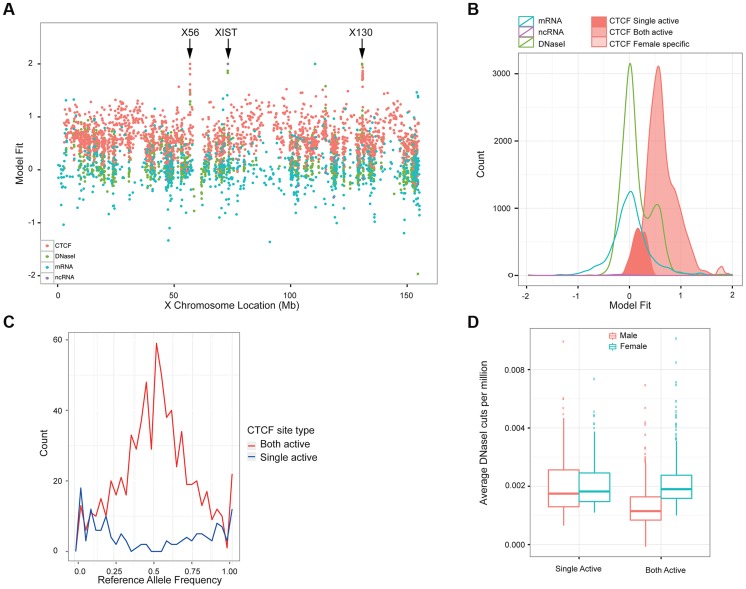
Properties of X chromosome CTCF sites. A. Plot of the metric to distinguish single-active from both active-sites, across the X chromosome for a variety of molecular assays (mRNA, ncRNA, DNase I and CTCF, coloured according to the key). B. A smooth density of the distribution of the dosage compensation fit for the 4 molecular assay types, with CTCF split into the 3 classifications (single active, both active and female specific). C. Allele-specific signal of heterozygote sites on the X chromosome from the 13 clonal female lines in the sample. The both-active sites show balanced allele-specificity, whereas the single-active sites show strong single allele CTCF binding. D. Box plot of the gender-specific behaviour of the DNase I assay at the major classes of X chromosome CTCF sites. DNase I data was collected in a different laboratory on different cell lines [17]. The both-active class shows a strong gender split, consistent with females having around double the signal, whereas the single-active sites show no gender change.

While exploring the correlation of between CTCF sites, we observed an unexpected behaviour of CTCF signal on the X chromosome. Strikingly, for 87% of CTCF sites on X (excluding the pseudoautosomal regions) there was a strong gender effect (P-value <0.01, Mann Whitney on gender); in nearly every case females have a significantly higher signal on average than males. The higher peak amplitude observed in females indicates, in effect, that the vast majority (87%) of CTCF sites on the X chromosome are occupied on both chromosomes. This is in contrast to the transcription of protein-coding mRNA (3% not compensated, i.e. X-inactivation escape genes), ncRNA (9%) [35] or other transcription factor occupancy as measured by DNase I (4%) (data from Degner *et al* [17]). We created a simple metric of the relative levels of activity, being the difference between the average male and average female signal, in each case adjusted for library depth as for the QTL analysis (Figure 5A and B). Protein coding mRNA and the majority of DNase I sites are consistent with only one active chromosome, leading to dosage compensated mRNAs(reviewed in [32]). As expected, there is a larger set of female specific ncRNAs, in particular the three XIST transcripts (Figure 5A). Using the Mann Whitney test of gender bias per site, we classified sites first as having significant bias, and then split the significant bias to cases consistent with balanced haploid behaviour, which we call “both-active” sites, and a small number of female-specific sites where there is a strong CTCF signal for females but almost no signal in males (Figure 5B). The remaining CTCF sites, which show similar levels between males and females we describe as single-active. The both-active sites and the single-active sites are evenly distributed along the chromosome (Figure 5A), and the XIST site and two clusters of female specific sites are obviously distinct from the rest.

We first confirmed that the single-active and both-active sites represent different modalities of CTCF binding, using intra-individual allele-specific analysis and independent DNase I data. LCLs are Epstein Barr Virus (EBV) transformed lines from a mixed B-cell population, and can be clonal or polyclonal, so that some female-derived LCLs show consistent or clonal X inactivation, whereas others have a mix of both X chromosomes being inactivated. Because we cannot assume that our LCL lines were clonal and therefore have consistent X inactivation, we first assessed the 17 female cell lines for clonal X inactivation status using heterozygous SNPs in genes known to be silenced on the inactive X [36], and selected the 13 lines with consistently skewed expression of these genes indicating consistent X inactivation (Methods). In these 13 lines, heterozygous SNPs in CTCF binding regions showed strikingly different behaviour between the single-active and both-active sites. The single-active sites showed strong allele-specific CTCF binding behaviour (similar to mRNA) whereas the both-active sites showed balanced signal over the two alleles from the very same samples (Figure 5C). In addition, we projected the DNase I data from the independent Yoruban cell lines onto the CTCF classification. For the 451 DNase I sites overlapping the CTCF sites on the X chromosomes, there was a strong concordance of this independent assay, performed on independent cell lines, with the classification of CTCF sites (Figure 5D). Both these analyses strongly support the finding that there are two major distinct types of CTCF binding sites on the X chromosome, with the both-active sites being bound on both the active and inactive X chromosome and the single-active sites being bound on only one chromosome (most likely the active X chromosome).

**Figure 6:**
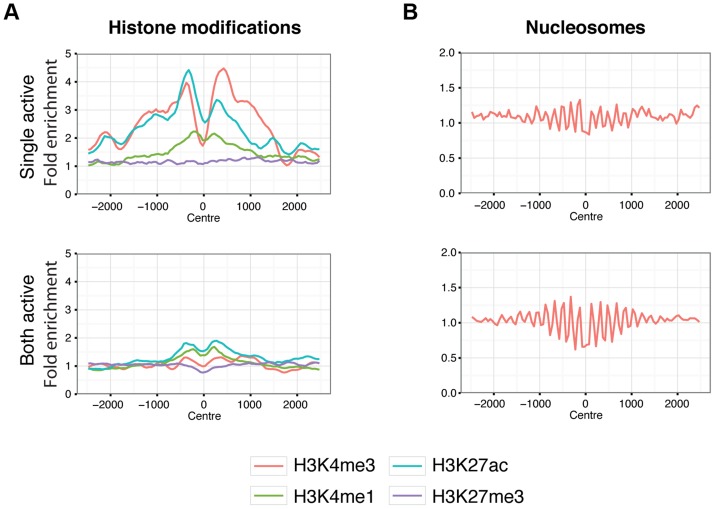
Chromatin behaviour around X chromosome sites. Aggregated signals for histone modifications (A.) and nucleosome positioning by micrococcal nuclease sensitivity (B.) at X chromosome binding regions split by single-active (top panels) and both-active (bottom panels) classes. Only binding regions distal to promoters are shown. Equivalent plots for binding regions including promoter regions are shown in Figure S19. Histone modifications are indicated by colour as described by the key below the plots. Signal data comes from GM12878 from the ENCODE project [37] and shows the average fold enrichment for this region against random Poisson distribution with local lambda.

We then explored differences between these two classes of CTCF sites, using the ENCODE data from the GM12878 LCL [37], derived from a female individual. The majority of histone modifications associated with active chromatin (H3K4me4, H3K27ac) showed strong enrichments in the single-active class of CTCF sites but not in the both-active class, even when we excluded promoters (Figure 6A). The repressive histone mark, H3K27me3, implicated in X chromosome inactivation, is similar between both classes of sites. Interestingly both classes showed nucleosome phasing (Figure 6B) albeit stronger at the both-active sites. There is not a striking change in Cohesin co-binding, as shown by overlap with Rad21 and SMC3 (Figure S20). The mammalian conservation of the two classes of CTCF sites is high and approximately similar (62% for single-active sites overall with GERP conserved elements, and 53% for both active sites), showing that both classes have been under selection across mammalian evolution. Overall there is strong evidence for a dramatic distinction of these two classes of sites in terms of local chromatin behaviour. When we considered histone marks from a smaller set of cell lines, but with a broader set of marks we do not observe the same set of gender-biased signals except for H3K27me3, consistent with it's role in X inactivation. (Figure S21).

**Figure 7:**
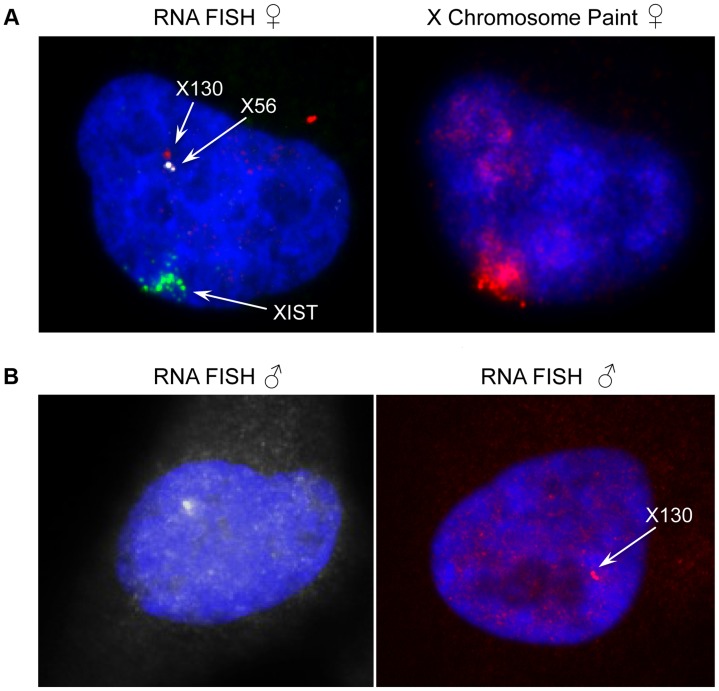
Expression and genomic organization of non-coding RNA genes X56 and X130. (A) Representative RNA-FISH image of X56 (white signal) and X130 (red signal) expression relative to XIST RNA (green signal) in female nuclei (counterstained with DAPI) and sequential DNA-FISH representative image with X chromosome paint probe (red signal) showing that X56 and X130 are transcribed from the active X chromosome nuclear territory. (B) Representative images of X56 (white signal) and X130 (red signal) expression by RNA-FISH in male nuclei (counterstained with DAPI).

We then turned to the 23 female-specific sites. These sites were concentrated in two loci overlapping non-coding RNAs (X56 and X130), largely identical to sites previously identified as being involved in a repeat-specific X chromosome behaviour [38]. Although there are far fewer sites to analyse than the other classes, the female specific sites are all enriched for binding to YY1, which is known to tether XIST to the inactive X nucleation centre [39]. Horakova *et al* [38] explored the RNA expression of these ncRNAs in female cells; we performed fluorescence *in situ* hybridization (FISH) for RNA in both male and female cells. Consistent with the published results [38], we detected RNA from the active X at these loci in female cells (Figure 7A). In male cells we also detected RNA expression (despite the female specific nature of the CTCF sites, Figure 7B), suggesting that these CTCF sites are likely to be involved in a female-specific inactivation process at these loci. Using the data from Kilpinen et al, we can show that these sites are active in female lymphoblastoid cell lines, but not male (Figure S23). It is notable how few of these sites there are on the X chromosome, compared to the far more numerous single-active and both-active categories.

We then turned to the 23 female-specific sites. These sites were concentrated in two loci overlapping non-coding RNAs (X56 and X130), largely identical to sites previously identified as being involved in a repeat-specific X chromosome behaviour [38]. Although there are far fewer sites to analyse than the other classes, the female specific sites are all enriched for binding to YY1, which is known to tether XIST to the inactive X nucleation centre [39]. Horakova *et al* [38] explored the RNA expression of these ncRNAs in female cells; we performed fluorescence *in situ* hybridization (FISH) for RNA in both male and female cells. Consistent with the published results [38], we detected RNA from the active X at these loci in female cells (Figure 7A). In male cells we also detected RNA expression (despite the female specific nature of the CTCF sites, Figure 7B), suggesting that these CTCF sites are likely to be involved in a female-specific inactivation process at these loci. It is notable how few of these sites there are on the X chromosome, compared to the far more numerous single-active and both-active categories.

## Discussion

This study is the first systematic association based analysis of how normal genetic variation in humans affects the binding of a sequence-specific transcription factor, where the binding is measured as a quantitative trait. The properties of the binding quantitative trait loci (QTLs) that we identified are consistent with and extend previous smaller-scale studies of how genetic variation affects CTCF binding [8], [12], as well as similar analyses of chromatin QTLs underlying DNase I hypersensitive sites [17]. We find a large number of QTLs, with the majority being within or close to the binding region, and approximately a quarter inside the bound CTCF motif. By using 1000 Genomes Project cell lines, we can be reasonably confident that we have a full catalog of common variation of which some subset are the causal variants. Using this information we could show that for a large fraction of the associations where the initial analysis suggested a distal variant more than 1 kb away, there was a plausible causal candidate also within 1 kb of the binding motif. Overall this suggests that, at least for CTCF, the substantial majority (∼75%) of common genetic variants in the region with a reasonably strong effect on its binding lie within 1 kb of the binding motif, although only a minority are actually within the motif. This clarifies previous observations that genetic variants contributing to transcription factor binding were typically not in the motif itself [9], [13] but there was enrichment nearby [11].

We see hundreds of sites showing allele-specific binding. The idea that allele-specific events have similar effects inside one cell as genotypic effects do between individuals is commonplace [40]. Here we show that these two effects are well modeled by a linear relationship (at least for this assay), though with an interesting subset of allele-specific sites that show no QTL. In contrast there are few QTL loci that overlap binding regions without an allele-specific signal.

As expected, some of the allele specific sites switch specificity between the alleles in different samples, consistent with a nearby, incompletely linked causal allele, random allelic inactivation or parent-of-origin imprinting. Many of these sites can be explained by an incompletely linked nearby locus, highlighting that the causal variant is often not co-incident with the binding region.

Finally with more confident mapping of reads from paired read ChIP-seq data we are able to show that a consistent signal towards reference alleles is in fact predominantly due to a biological effect favouring ancestral alleles (at least for the CTCF transcription factor). This suggests that base pair changes segregating in the population tend to reduce binding of existing sites (rather than create new sites), at least for CTCF, and this is consistent with CTCF motif creation occurring by non-base pair changes, e.g. repeat deposition, as suggested in Schmidt *et al* [41].

We were initially surprised by the strikingly different behaviour of CTCF on the X chromosome compared to gene expression. Unlike transcribed genes, a large proportion of CTCF sites behave in a similar manner on both chromosomes. This is due to the same sites being bound on both the active and inactive X chromosome in females, as shown by the distribution of CTCF signal, the corresponding change in DNaseI signal in entirely separate cell lines and the lack of allele-specific signals in heterozygote sites in this class. This suggests that there is a subset of CTCF sites on the X chromosome that is bound on both copies despite the striking large scale compaction of the inactive X. This X chromosome-wide behaviour of CTCF is a very different phenomenon to the locus-specific interaction at the Xist/Tsix locus implicated in determining which X chromosome is inactivated [42], [43].

This observation has a number of implications. It is consistent with the multi-functional nature of CTCF, which has been commented on many times before in locus-specific [20]–[26] or specific genome-wide screens [27]–[30]. In this study we only examined behaviour in lymphoblastoid lines, and there might be cell type specific differences as well. Single-active sites show histone modifications and TF co-binding consistent with involvement with regulating expression on active chromatin. In contrast, the both-active sites show far less complex histone modification, consistent with structural functions that might apply to both chromosomes. Finally although we discovered this phenomenon on the X chromosome due to how these sites interact with X chromosome inactivation, it is consistent with the different binding behaviours of CTCF seen on the autosomes, with a diversity of different histone modification patterns at different CTCF sites [37].

The female-specific CTCF sites on the X chromosome are a very distinct subset; these are placed mainly over two non-coding RNAs expressed from the active X in females and males. The simplest explanation is that CTCF binding at these sites is involved in transcription repression on the inactive female X chromosome. This catalog of CTCF QTL sites is part of a growing set of molecular assays that are being examined in outbred individuals (for example, see [12], [17], [40], [44], [45]). It provides a specific hypothesis for the 63 disease related loci which overlap these QTLs, and for future overlaps with other molecular, cellular and disease related phenotypes. The gradual unraveling of the different variant effects on different molecular behaviour will provide a growing understanding of molecular and physiological processes in health and disease.

## Materials and Methods

### ChIP-seq

Cells were cross-linked with 1% formaldehyde for 7 min at room temperature. Formaldehyde was deactivated by adding glycine. Chromatin from harvested cells was sonicated with a Bioruptor to an average size of 500 bp DNA. Immunoprecipitation was performed using sonicated chromatin by adding anti-CTCF antibody (Millipore 07-729). ChIP DNA was used to generate a ChIP-seq library according to the standard Illumina protocol. The library was then sequenced using the Illumina HighSeq platform in 50 bp paired end reads. On average ∼85.54M reads were produced per sample. Sequence lanes were assessed for multiple quality metrics including total yield, read quality, mapping quality, GC content distribution and duplication rate. All sequencing reads were aligned to the human reference sequence (GRCh37) using BWA v0.5.9-r16 [46] using default parameter settings. Duplicate reads were marked by the “MarkDuplicates” function of the software Picard (v1.47 http://picard.sourceforge.net/) and removed. We applied a stringent filter by removing all the reads with MAQ quality score below 30, improperly paired (with 0x2 flag set in the BAM format), or with mate pairs more than 1 kb apart were removed. For allele specific analysis, we further performed local realignment using a variant-aware aligner glia (https://github.com/ekg/glia), which aligns reads against paths in a variant graph built by combining the reference sequence and known variants.

### Data processing

#### Genotypes

Our 51 samples consist of 35 individuals present in the 1000 Genomes Phase 1 release (v3 20101123) [2], 11 individuals in the 1000 Genomes Pilot, 2 individuals in 1000 Genomes high coverage Trio (NA12891 and NA12892) and 3 individuals in the HapMap III [47]. The eleven 1000 Genomes Pilot samples have low coverage. We calculated the genotype likelihood for each of the Phase 1 sites using samtools [48] and then performed imputation using BEAGLE [49] and IMPUTE2 [50] with the 1000 Genomes Phase 1 data as a reference panel. Using Illumina Omni 2.5M SNP array genotypes (available from ftp://ftp.1000genomes.ebi.ac.uk/vol1/ftp/technical/working/20120131_omni_genotypes_and_intensities/) as a validation set, we obtained good accuracy from this procedure with a mean non-reference discordance rate of 2.33% and average genotype dosage R^2^ of 0.956. We also imputed the three HapMap III samples, using their genotype data on the Omni 2.5M array as the imputation panel and the 1000 Genome Phase 1 as the reference panel. We then integrated data from each source and obtained a consolidated genotype set for all 51 individuals. For association mapping, we filtered variants by requiring >5% minor allele frequency, P value for Hardy-Weinberg Equilibrium (HWE)>1E-4 and position within 50 kb of the binding region being mapped. Finally, 4,687,317 variants entered analysis, with 4,250,881 SNPs and 436,436 INDELs.

The 1000 Genome Phase 1 release gives a comprehensive ascertainment of the genetic variants. However, it is still possible that some variants private to this study cohort are yet to be found. To address this concern, we performed variant calling for the CTCF binding regions using ChIP-seq data. The calling was done by using samtools mpileup with parameters “-DV -C50 -q 30 -Q 30 -d 10000 –u -l $qtl_regions -b $bam_list -f $reference”, followed by BCF tools with parameter “-t $qtl_regions -mv”. We filtered on the quality of the calling by keeping only variants with QUAL score greater than 20. We also kept only the variants that are private to the new call set and are absent in the 1000 genomes phase1. In the end, we obtained 4,756 variants are within binding regions with 2,282 SNPs and 2,474 INDELs. It is a small quantity compared to the variant set of the 1000 Genome Phase1 release. This set is also enriched for INDELs (52%). When we conducted the same association scan using these variants, we discovered 55 QTL binding regions associated with 60 variants, out of which only 8 are new. We also repeated the same analysis but with a lower threshold for the mapping quality, the results are similar with very marginal increase in findings. Thus the effect of this additional variant set is minimum in our QTL scan.

#### Binding region calling

We performed binding region identification using a Parzen kernel density window algorithm described previously [18], [51]. This procedure was applied to both experimental and Input datasets after combining lanes and replicates into cell-line sample sets. Local maxima of these Parzen scores were used to define binding peak positions, and the interquartile range of the kernel density profile was used to determine the corresponding binding site of highest read density. The resulting set of candidate CTCF binding sites was then subjected to input correction, filtering for copy number artifacts, and determination of statistical significance.

First, in order to normalize for background represented by the Input control, each binding site was paired with the corresponding Input site with the highest read count within 200 bp. A binomial P-value was computed for each binding site under the null hypothesis that ChIP and Input reads were equally likely. The ratio of total ChIP to Input reads for each sample was used to normalize for differences in sequencing depth before calculating the binomial *P*-value, with the library having higher sequencing depth always scaled downward. Binding sites falling in previously defined genomic regions with aberrantly high signal due to copy number differences were discarded [5]. Input-dominated binding sites were also discarded, retaining only sites where the sequencing-depth-scaled ChIP read count exceeded Input.

The resulting set of filtered peak P-values was subjected to multiple hypothesis testing using the Benjamini-Hochberg method [52]. Next, binding regions for the cell lines at various significance levels were merged using bedtools v2.17.0 [53] in such a way as to preserve the set of calling cell lines (bedtools merge -nms -scores collapse -n). We employed several metrics in order to determine an appropriate significance cutoff, including the relationship between binding region count and P-value (Figure S15) and the number of calling cell lines for each binding region (Figure S16). binding regions with BH-adjusted P-value ≤1E-5 were initially retained as significant (n = 127,351), as that value appeared to be the inflection point in the binding region versus P-value curve and had the largest reduction in one-caller binding regions.

Finally, in order to assess the quality of binding regions called by only one cell line, we used bedtools (bedtools intersect –c) to identify binding regions containing the extended CTCF motif (Figure S17). binding regions called by only one cell line showed a significantly lower occurrence of the CTCF motif as compared to binding regions called by two or more cell lines. Therefore, we discarded binding regions with only one calling cell line and retained the 63,753 merged binding regions at adjusted P-value 1E-5with two or more callers.

#### Blacklisting regions

Out of 63,753 binding regions identified, we removed 2,898 binding regions falling in repeat sequences or in the Immunoglobulin heavy chain locus or major histocompatibility complex (MHC). In detail, 2,578 binding regions lie completely within repeat sequences marked by a merged set consisting of “Repeat Masker”, “Segmental Dup” or “Simple Repeat” data sets from the table browser of the UCSC Genome Browser, 35 binding regions lie within the Immunoglobulin heavy chain locus (chr14:106053226–106330470) and 285 fall in the MHC region (chr6:28477797-33448354).

#### Motif word identification

We searched for the instances of CTCF motif in the discovered binding regions using the CTCF canonical 19 bp position weight matrix downloaded from the JASPAR database (http://jaspar.binf.ku.dk/). We extracted DNA sequences at the identified binding regions from human genome reference GRCh37 to construct a sequence database. The search was then performed using the software FIMO [54] in the MEME tool suite [55] using parameter –threshold 1E-4. This process identified at least one motif instance in 45,867 of our 57,428 binding regions. When calculating the overlapping between QTL variants and motifs we considered all discovered motif instances within a binding region. For the QTL variants that do not overlap any motif variants, we used the motif instance within binding region with the best matching score as an anchor for calculating the distance.

#### Identifying motif of other factors

We also searched for other motifs at regions nearby (+−30 bp) the lead QTL variants as well as within the QTL binding regions. We extract sequences of the regions using software *fastaFromBed* of the BEDTools suite [53] using reference sequence GRCh37. We then used software MEME [55] to search for motifs. The discovered motifs were then compared against known motifs using software tomtom [55] in JASPAR and UniPROBE databases. The discovered motifs are then provided to MSigDB [56] for functional annotations.

The most enriched motif was a CTCF related motif. After removing a number of low complexity motifs and cryptic repeats by manual inspection, the next most enriched motif was a G-rich motif similar to multiple SP-1 sites. There was not a strong accessory motif in these regions.

#### CTCF binding quantitation

With the peak profile identified above, we quantified the signal for each binding region by counting the number of sequencing fragments - read pairs. We applied stringent criteria by only counting the properly aligned read pairs with quality score at least 30 and excluding all the duplicated reads(samtools view -f 0x42 -F0x604 –q 30). We used Bedtools (v2.16.2) [53] to count the intersection between fragments and identified binding regions. This produced an *N* by *M* matrix, where *N* is the number of samples and *M* is the number of binding regions. To evaluate the variation in the ChIP experiments, we compared the correlation between replicates grown on consecutive days and the correlation between all other samples. We found a mean pairwise correlation coefficient of 0.8314±0.0006 and 0.8202±0.0099 for the replicate sets for NA12891 and NA12892, respectively, while the mean pairwise correlation coefficient between samples was 0.1719±0.0177. This suggests a good signal noise ratio in the experiment.

For the subsequent genetic analysis, we are interested in the binding regions that have good signal and also vary between individuals. The mean and variance of binding intensities are correlated by the nature of the Poisson process for the sequencing. We found a group of 4,516 binding regions (7% of the total binding regions identified) with little signal or variation - defined as binding regions mapped with fewer than 6 fragments on average per sample and SD<5.14 (Figure S18). These binding regions were excluded from further analysis.

#### Normalization

Previous studies [17], [31] have shown that appropriate normalization can substantially enhance genetic association signals by removing confounding non-genetic sources of variation. Potential sources of confounding variation include experimental batch effects, GC bias in sequencing library construction and latent unknown technical or biological factors that have systematic effects across large numbers of binding regions. To address these issues, we normalized the raw binding intensity using the following five step approach to generate a normalised adjusted binding intensity (NABI).

1. Rescale by sequence depth.
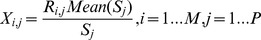
where *R_i,j_* is the raw intensity of the *i*th binding region of the *j*th lane, and *S_j_* is the sum of intensity across all binding regions for the *j*th lane. *R_i,j_* is scaled by a factor of the proportion of mean of *S* across all P lanes over *S_j_*.
2. Remove variance introduced by GC composition. We adjusted for GC bias in sequencing library construction by forming percentile bins for GC composition of all binding regions and normalising the binding intensities within each bin.
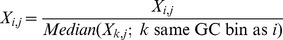
where *i,j,k* are the indices for binding region, lane, and GC bin respectively.
3. Merge lanes of a same individual by taking the mean. A subset of our samples were sequenced on multiple lanes and in these cases we took the mean value across lanes as the measurement of the individual.
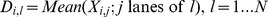
where *X_i,j_* is the measure from the previous step, *i,j,l* are indices for the binding region, lane and samples, respectively. *N* is the total number of samples.
4. Centre-scale binding intensity for each binding region. We then scaled the binding intensity for each binding region by subtracting the mean and then dividing by the standard deviation. This transforms the measures of each binding region into zero mean and unit variance, which is needed for the quantile normalization to be less affected by the different variances of different binding regions
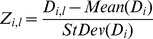
where *i,l* are indices for binding region and sample.
5. Quantile normalize each sample data to a normal distribution. The distribution of binding intensities for each individual is complex. Previous studies have shown that quantile normalization, initially developed for normalising the microarray signals of gene expression, can assist statistical analysis by converting the distributions of each sample to a reference distribution. The linear regression model used to identify QTL in our study assumes a Gaussian distribution of binding measures within each genotype class. We therefore mapped the measures across all binding regions of each sample to the corresponding normal quantiles. This produces a matrix that is essentially a perturbation permutation of the normal quantiles
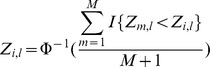
where Φ is the cumulative normal density function and *M* is the total number of binding regions. *I* is an indicator function that returns 1 if the condition is met and 0 otherwise.
6. Remove confounding variation by principal component analysis (PCA). The measures of binding for each individual can be confounded by a number of hidden factors due to either biological or technical factors, or both. We performed PCA and saw that the first factor explained 24.1% of the variance in the data, substantially more than later components (Figure S2). Further investigation of this component showed that it was correlated with ChIP batch date, and it was therefore removed.

#### Association and estimation of the false discovery rate (FDR)

We applied linear regression for association testing. For each binding region, we tested the association between the binding intensities and the genotypes of the variants that are within ±50 kb of the binding region by least-squares linear regression. We applied an additive model, where genotypes are coded as the dosages of the alternative alleles, either 0,1, or 2. The P value was obtained from a t-test of the regression coefficient (beta) against the null hypothesis of beta = 0. We estimated FDR by a Q value method [34], which establishes P<7.1E-5 as an FDR of 1%. We further filtered the associated SNPs by requiring the P value to be within 1 order of magnitude to that of the P value of the lead SNP. We report these cluster variants as associated to the target binding region. We also reported results when a more stringent Bonferroni threshold was applied. The threshold was calculated at a significant level of α = 0.05 corrected for 13,293,727 tests, which gives 3.8E-9 for the actual threshold.

### Allele-specific analysis

Read counts at each allele were counted for the 5.6M SNPs within 50 kb of a binding region. Heterozygous SNPs with significant allele-specific CTCF binding were identified. In detail, we calculated a binomial P value at all heterozygous SNPs with the null hypothesis that the two allele counts are equal. We then performed multiple testing adjustment at all heterozygous SNPs that have at least 2 reads at each allele and at least 2 reads difference between the two alleles using the Benjamini & Hochberg [52] method. Significant allele-specific binding was determined with an FDR 5%.

### X chromosome analysis

We analysed the gender specific CTCF binding on the X chromosome in the 27 female and 24 male LCLs. To ensure that our normalisation would not introduce any bias we used the raw CTCF binding intensities. For each of the 1,968 binding regions on the X chromosome, after blacklisted regions were removed, we assessed gender specificity by a Mann-Whitney U (MWU) test between the male and female samples. Binding regions were then classified as single-active and both-active based on the significance of the MWU test on the binding intensities. To classify the female specific binding regions we also incorporate the fold change between the average male and average female binding intensity. Similar analysis as for CTCF was performed on mRNA and ncRNA data from the Geuvadis project [35] and on DNase I [17].

To differentiate clonal and ployclonal sample, we analyzed allelic RNA expression on previously identified X inactivated genes [36] in 17 female samples. Samples where only one allele is expressed are determined to be clonal and polyclonal samples have RNA expressed from both alleles. Figure S22 shows examples of a clonal and a polyclonal sample.

For the single-active and both-active sites, we analysed the overlap of each category of sites with the ENCODE transcription factor and histone modification datasets for the female CEU lymphoblastoid cell line GM12878 [37]. To avoid bias introduced by unequal distribution of promoter sequences between the classes, we removed all binding regions that overlap with promoters identified in GM12878. For each binding region we define a partial overlap as an overlap. Signal aggregation of each of the classes of sites for histone modification and TF ChIP-seq data, and micrococcal nuclease cleavage was calculated using the ACT toolkit (http://act.gersteinlab.org/, [57]) with the parameters ‘-nbins = 50 -mbins = 0’. Only binding regions that are in the top 50% of bound sites were used. ENCODE bedGraph files for both TF and histone modifications were obtained from ftp.ebi.ac.uk: pub/databases/ensembl/encode/integration_data_jan2011/byDataType/signal/jan2011/bedgraph/and converted into signal files that are used as input for ACT.py.

### Sequential RNA and DNA fluorescence in situ hybridisation (FISH)

Female and male human dermal fibroblasts cells (Invitrogen) were grown directly on Nunc Lab-Tek chamber slides, rinsed briefly using 1×PBS (PAA) and immediately fixed in 3% formaldehyde (Sigma-Aldrich) for 10 minutes, permeabilized using 0.5% Triton X-100 (BHD), 10 mM Ribonucleosidase Vanadyl complex (Biolabs) in 1×PBS (PAA) for 10 minutes, and then dehydrated through a 70%, 90% and 100% ethanol series, all at room temperature. The probes for X56 and X130 were selected according to their genomic locations reported in Horakova *et al* [38]. The probe for X56 consisted of the BAC clone RP11-416J22 and the fosmid G248P8472H8, the probe for X130 consisted of the BAC clone RP11-158M12, while the probe for XIST consisted of the fosmid G248P8779H11. All the clones were selected from the UCSC Genome Browser (GRCh37/hg19 assembly). Plasmid DNA was purified using the PhasePrep BAC DNA kit (Sigma-Aldrich) following manufacturer's protocol, amplified using the whole genome amplification kit (WGA2, Sigma-Aldrich) following manufacturer's recommendations. Clones were labeled using the whole genome re-amplification kit (WGA3, Sigma-Aldrich) as described before [58]. Briefly, X56 probe was labeled with Cyanine 3-dUTP (Enzo), the X130 probe was labeled with ChromaTide Texas Red-12-dUTP (Invitrogen) and the XIST probe was labeled with Green-dUTP (Abbott). For RNA-FISH, approximately 100 ng of labeled DNA from each probe and 2–4 µg of human Cot-1 DNA (Invitrogen) were ethanol precipitated, then resuspended in hybridisation buffer containing 50% formamide, 2×SSC, 10% dextran sulphate, 0.5 M phosphate buffer, pH 7.4. The probe mix was denatured at 65°C for 10 minutes before being applied onto cells on the chamber slides. Hybridisation was carried out in a 37°C incubator overnight. The post-hybridisation washes consisted of two rounds of 50% formamide/2×SSC washes followed by two additional washes in 2×SSC. All washes were done at 40°C, for 5 minutes. After detection, slides were mounted with SlowFade Gold mounting solution containing 4′,6-diamidino-2-phenylindole (Invitrogen). Images were visualised on a Zeiss AxioImager D1 fluorescent microscope. Digital image capture and processing were carried out using the SmartCapture software (Digital Scientific UK).

For the subsequent DNA-FISH, the same slides that have passed through the RNA-FISH assay described above were subject to the following treatment before denaturation in 70% formamide/2×SSC for 1.5 minutes, including one wash in 2×SSC for 5 minutes, digestion with RNase A (100 µg/ml RNase A in 2×SSC) for 30 minutes at 37°C, further digestion with 0.01% pepsin in 10 mM HCl for 5 minutes at room temperature, dehydration through an ethanol series as above and ageing on a 65°C hot plate for an hour. The X chromosome paint probe was labeled with biotin-16-dUTP (Roche). The making and denaturation of the X chromosome paint probe mix, hybridisation incubation, post-hybridisation washes and digital imaging were the same as above described, except that the biotin-labeled probes were visualised using Cy3 conjugated avidin (Sigma Aldrich).

### Accession numbers

The ChIP-seq data reported in this paper have been deposited in the European Nucleotide Archive, available with accession number ERP002168. The sample information and experimental design was deposited in ArrayExpress with accession number E-ERAD-141, linked to ERP002168.

## Supporting Information

Figure S1 Higher correlation within day replicates compared to between different samples. We calculate the pair-wise Spearman correlation among all samples, including the two day-replicates, 12891 and 12892, shown as the last two sets of four samples. A diagonal line in each cell represents perfect correlation whereas a full circle represents no correlation. Increasingly flattened ellipses indicate a greater degree of correlation. When comparing among the day replicates, we obtained a correlation coefficient of 0.8314 and 0.8202 for GM12891 and GM12892, respectively. We also looked at the mean correlation of all the other samples and found a correlation of 0.1719. Therefore we see much higher correlation within day replicates than that of all other samples.
(PNG)

Figure S2 Proportion of phenotypic variance explained by each principal component (PC). We performed principal component analysis (PCA) on the normalized data to discover latent factors that explain large proportion of phenotypic variation. We saw that the first principal component explain substantially more variance than the others. When we looked at the correlation between the first principal component and technical and experimental variables, we found that it correlates with ChIP batch at ρ = 0.47. The first principal component is removed from the data before further analysis.
(PDF)

Figure S3 The number of significant QTLs found as a function of false discovery rate (FDR), plotted for the raw data and after each stage of the data normalization procedure that we used (see Methods for details of the method). We first normalised the binding intensities for each sample by the total read depth for that sample. We then corrected for GC composition by removing the median count of binding regions in the same GC bin (100 bins in total) from each binding region. The measures for each binding region were then centre-scaled by removing the mean and then dividing by the standard deviation (track hidden behind GC as center scale does not affect regression). This was followed by a quantile normalization, which maps the measures of each sample to normal quantiles across all binding regions. Lastly, we removed the first principal component that explains the most global phenotypic variation.
(PDF)

Figure S4 QQ plot for all associations between CTCF binding intensities and genotypes of variants within 50 kb to the centre of binding sites. Purple and green dots indicate P values from actual tests and permutation controls - where sample labels are randomly permuted. We used 1% FDR (brown line) as our cutoff for results.
(PDF)

Figure S5 Spearman rank test for association is more conservative but gives similar results. Association test by linear methods can be inappropriate and gives spurious signal if the normality assumption is not met. Although in our normalization procedure the binding measures are mapped to normal quantiles sample-wise, it is still possible that the normality assumption does not hold binding region-wise. To test if this would bias the QTL mapping we performed the same tests using the Spearman rank method. The P values from both sets are sorted and then plotted against each other as Y-axis for the linear test and X-axis for the Spearman rank test. We see a slight elevation of the black line, suggesting the rank test is more conservative but would give similar results, and our linear test is mostly appropriate.
(PNG)

Figure S6 P value distribution of the proximal variants. Here the P values from the association between the CTCF binding and the lead distal QTL variants are plotted against that of the proximal variants, which are in LD with the distal QTL variants. The horizontal and vertical dashed lines are the 1% genome wide FDR threshold established in the main analysis. The diagonal line assists to indicate same P values. Each dot is colored by its D′ value of LD with its size scaled by the allele frequency of the proximal variant.
(PDF)

Figure S7 Distribution of the proximal variants that are on motif and in LD with the distal lead QTL variants. Here the proximal variants were aligned to the motif positions. We saw a correlation between their distribution and the information content of the motif at ρ = 0.36.
(PDF)

Figure S8 Evidence for indirect effects when a second binding region is present in the distal QTL window. Many (75.5%) of our distal QTLs contain a second CTCF binding region in their 50 kb *cis*-window. To explore possible causal relationships between the lead variant, the associated binding region(BR1) and the second binding region(BR2) we constructed seven graphical models (A) and compared them using the Bayesian Information Criterion (BIC). In each case we assign the most likely model, chosen as having the lowest BIC. The frequency of the chosen models (B) suggests that there is almost never evidence for the association effect of the distal variant being mediated via a secondary binding region. The most frequently preferred model (1) did not involve BR2 at all; for the next most preferred models (3 and 4) there was some evidence of interactions between neighbouring CTCF binding sites, but we could not explain the variant association to BR1 binding via BR2. The only models which support mediation of binding at BR1 via BR2 are 5 and 6, and in only one case do we see one of these being selected. The P value of BR1 when conditioned on BR2 is plotted in (C). We further investigated the enrichment of a range of ENCODE [1] signals over the QTL binding region and the neighboring region. We found the association between two binding regions (model 3,4) tend to correlate with the active regulatory signals (Figure S9).
(PDF)

Figures S9 The interaction between QTL binding region and neighboring binding region correlates with regulatory events. The distal QTL set is as previously described (Figure S8). For each of the four categories with sufficient abundance (model 1, 2, 3 and 4), we compare the average signals between the QTL binding region (B1) and the neighboring binding region (B2) for a number of molecular markers using data obtained from the ENCODE project [1]. We observed distinct patterns of regulatory signals between model 1,2 and model 3,4. We saw that when there exists interactions between two binding regions (model 3,4), active transcription factors, enhancers and active histone markers tend to be more enriched in the QTL binding regions, as shown in red. This change is not driven by their distances being closer to the transcription start site (TSS) by chance, measured as the distance to the closest TSS, because the neighboring binding regions have similar distance to the TSS as the QTL binding regions (red and green lines in the density plots). Some of the histone modifications (H2AZ, H3k27ac, H3k4me1, H3k4me2 and H3k4me3) swap enrichment direction between model 3 and model 4 depending on the direction of interaction between B1 and B2 (also see Figure S10 for more detailed enrichment signals).
(PDF)

Figure S10 Change of histone modifications depending on the interaction models between the QTL binding region and the neighboring binding region (see Figure S8 and S9 for explanations about the models).
(PDF)

Figure S11 Effect size versus derived allele frequency for all CTCF QTLs identified at 1% FDR.
(PDF)

Figure S12 Effect of the Reference Allele. Even when the reference allele is the derived allele (Derived), the binding bias remained towards the ancestral allele.
(PDF)

Figure S13 Effect of alignment to allele specific analysis. We performed local realignment using a variant aware aligner glia (https://github.com/ekg/glia) and compared the allelic bias in our significant allele specific sites between the two alignments. We saw that the effect of local realignment is minimum.
(PDF)

Figure S14 No QTLs with strong effect size in binding regions that do not show strong allele specificity. The x-axis shows allele specificity (measured as % reference), and the y-axis shows between-individual effect (beta) orientated such that positive is towards reference.
(PDF)

Figure S15 Number of merged binding regions plotted as a function of −log(BH-adjusted binomial P-value).
(PDF)

Figure S16 Number of merged binding regions as a function of number of calling cell lines, at three adjusted P-values.
(PDF)

Figure S17 Proportion of merged binding regions as a function of number of calling cell lines, at three adjusted P-values.
(PDF)

Figure S18 Quality control by raw signal intensity and inter cell line variability. For each binding region we counted the overlapping sequencing fragments (identified by a properly paired read pair) and used it as a measure for the raw binding intensity. We plot the log of the variance of the binding intensities across 51 individuals versus the log of the mean of the binding intensities using the R function *smoothScatter*. The degree of blue is proportional to the density of data points. As a Poisson process the mean and variance correlate with each other. There exists a natural cut-off between the lower left tail and the majority at mean 6 and standard deviation 5.14. These lower left tail binding regions are the sites with very low intensity and also low variability. We removed these sites, 4,516 binding regions in total, before further analysis.
(PDF)

Figure S19 Aggregated signals for histone modifications at X chromosome binding regions split by single-active (top panels) and both-active (bottom panels) CTCF classes for binding regions overlapping promoters. The average ENCODE signal, in GM12878, is determined by the average fold enrichment for this region against random Poisson distribution with local lambda [2].
(PDF)

Figure S20 Aggregated signal for transcription factors SMC3 and Rad21 at X chromosome shown for single active and double active binding regions. We plot the aggregated average ENCODE signal in GM12878, which is determined by the average fold enrichment for this region against random Poisson distribution with local lambda [2]. Single active CTCF sites tend to have a small increase in binding.
(PDF)

Figure S21 Gender difference in transcription factor binding and histone modification. Using data from [2] (5 males and 5 females, all unrelated), we compared the average signals between males and females on the X (black) and autosome (blue) for a range of markers with data obtained from the ENCODE project [1]. For each marker, all data is used, irrespective of overlapping with CTCF binding. Mann Whitney test is performed separated data on gender. A significant Mann Whitney test indicates a gender specific marker binding. We observed minimum gender specific signals, except for H3K27me3.
(PDF)

Figure S22 Examples of clonal and polyclonal cell lines. X chromosome genes are grouped according to their expression on the inactivated X [3]. On the x axis, 0/9 are the most strictly X inactivated genes and 9/9 are the genes that show consistent expression from the inactivated X. ‘NA’ denote the genes whose X inactivation status was not determined. On the y axis, percent reference reads from RNA-seq data were counted on the heterozygous SNP sites within those genes and plotted against their X inactivation status. NA12749 on the left was determined to be a clonal sample and NA12761 on the right was determined to be a polyclonal sample.
(PDF)

Figure S23 Aggregated signal for transcription factors using data from [2] (5 males and 5 females, all unrelated). We plot the average raw read signal, for several markers in regions that overlap CTCF binding region. Aggregate plots are separated on gender and CTCF classification. We observe that for the both-active and single-active CTCF sites there is, as expected, double as much signal for female than for male cell lines. For regions that show female specific CTCF binding, the aggregated signal track show a change in binding profile for H3K4me3, H3K4me1, and H3K27ac.
(PDF)

Table S1 Sites with random allelic bias. See Supplementary MS Excel file “switching_sites.xlsx”.
(XLSX)
