# Correction: Quantitative Genetics of CTCF Binding Reveal Local Sequence Effects and Different Modes of X-Chromosome Association

**DOI:** 10.1371/journal.pgen.1005177

**Published:** 2015-04-28

**Authors:** Zhihao Ding, Yunyun Ni, Sander W. Timmer, Bum-Kyu Lee, Anna Battenhouse, Sandra Louzada, Fengtang Yang, Ian Dunham, Gregory E. Crawford, Jason D. Lieb, Richard Durbin, Vishwanath R. Iyer, Ewan Birney

The final paragraph in the Results section is duplicated. The first version of the paragraph is correct and is provided here.

We then turned to the 23 female-specific sites. These sites were concentrated in two loci overlapping non-coding RNAs (X56 and X130), largely identical to sites previously identified as being involved in a repeat-specific X chromosome behaviour [38]. Although there are far fewer sites to analyse than the other classes, the female specific sites are all enriched for binding to YY1, which is known to tether XIST to the inactive X nucleation centre [39]. Horakova et al [38] explored the RNA expression of these ncRNAs in female cells; we performed fluorescence in situ hybridization (FISH) for RNA in both male and female cells. Consistent with the published results [38], we detected RNA from the active X at these loci in female cells (Fig 7A). In male cells we also detected RNA expression (despite the female specific nature of the CTCF sites, Fig 7B), suggesting that these CTCF sites are likely to be involved in a female-specific inactivation process at these loci. Using the data from Kilpinen et al, we can show that these sites are active in female lymphoblastoid cell lines, but not male (S23 Fig). It is notable how few of these sites there are on the X chromosome, compared to the far more numerous single-active and both-active categories.
