# Supplementary figures and images for: Quantitative Genetics of CTCF Binding Reveal Local Sequence Effects and Different Modes of X-Chromosome Association
Source: PLoS Genet. 2014 Nov 20;10(11):e1004798. doi: 10.1371/journal.pgen.1004798 (PMC4238955; doi:10.1371/journal.pgen.1004798)

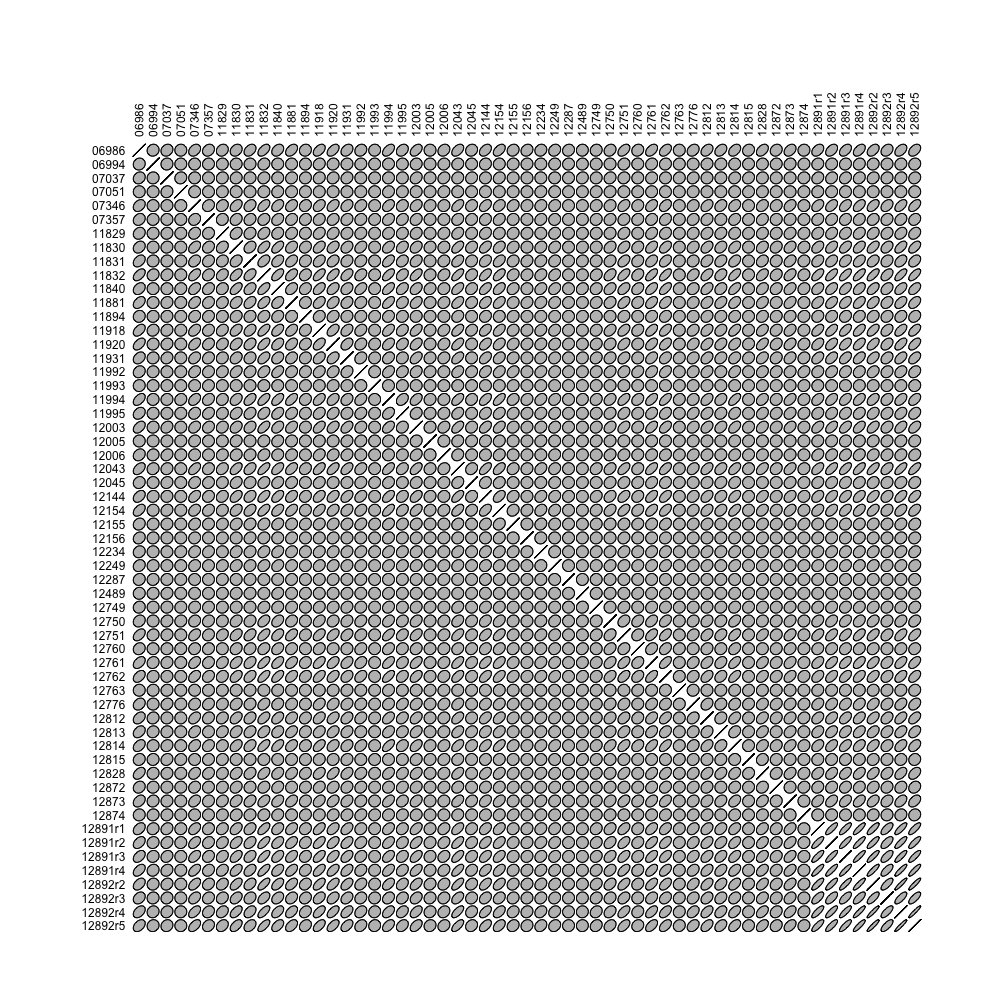

Supplement: Figure S1 — Higher correlation within day replicates compared to between different samples. We calculate the pair-wise Spearman correlation among all samples, including the two day-replicates, 12891 and 12892, shown as the last two sets of four samples. A diagonal line in each cell represents perfect correlation whereas a full circle represents no correlation. Increasingly flattened ellipses indicate a greater degree of correlation. When comparing among the day replicates, we obtained a correlation coefficient of 0.8314 and 0.8202 for GM12891 and GM12892, respectively. We also looked at the mean correlation of all the other samples and found a correlation of 0.1719. Therefore we see much higher correlation within day replicates than that of all other samples. (PNG) [file pgen.1004798.s001.png]

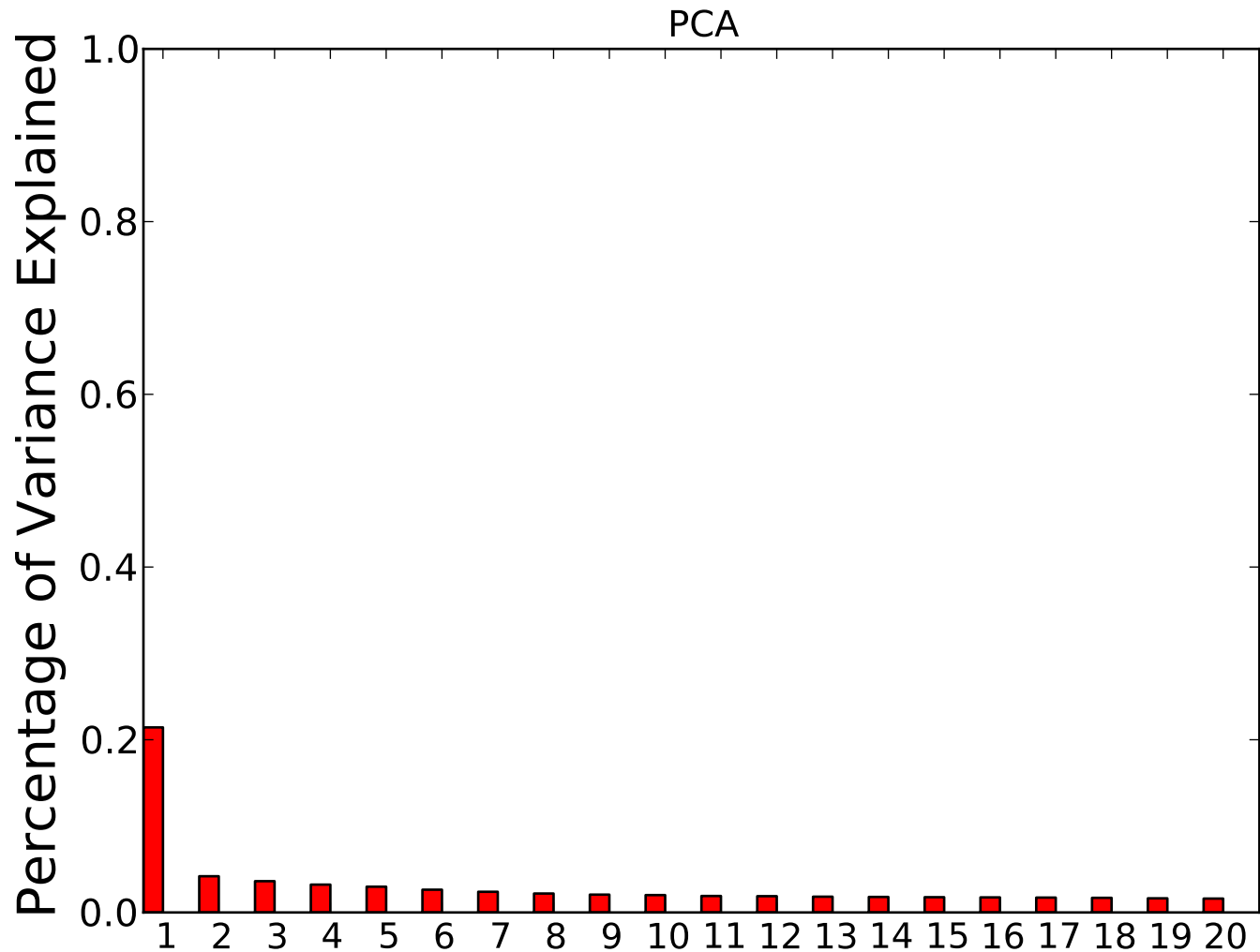

Supplement: Figure S2 — Proportion of phenotypic variance explained by each principal component (PC). We performed principal component analysis (PCA) on the normalized data to discover latent factors that explain large proportion of phenotypic variation. We saw that the first principal component explain substantially more variance than the others. When we looked at the correlation between the first principal component and technical and experimental variables, we found that it correlates with ChIP batch at ρ = 0.47. The first principal component is removed from the data before further analysis. (PDF) [file pgen.1004798.s002.pdf]

# of Significant variants

FDR

Step

- PCA 1PC
- Quantile
- GC
- Centre Scale
- Read Depth
- Raw

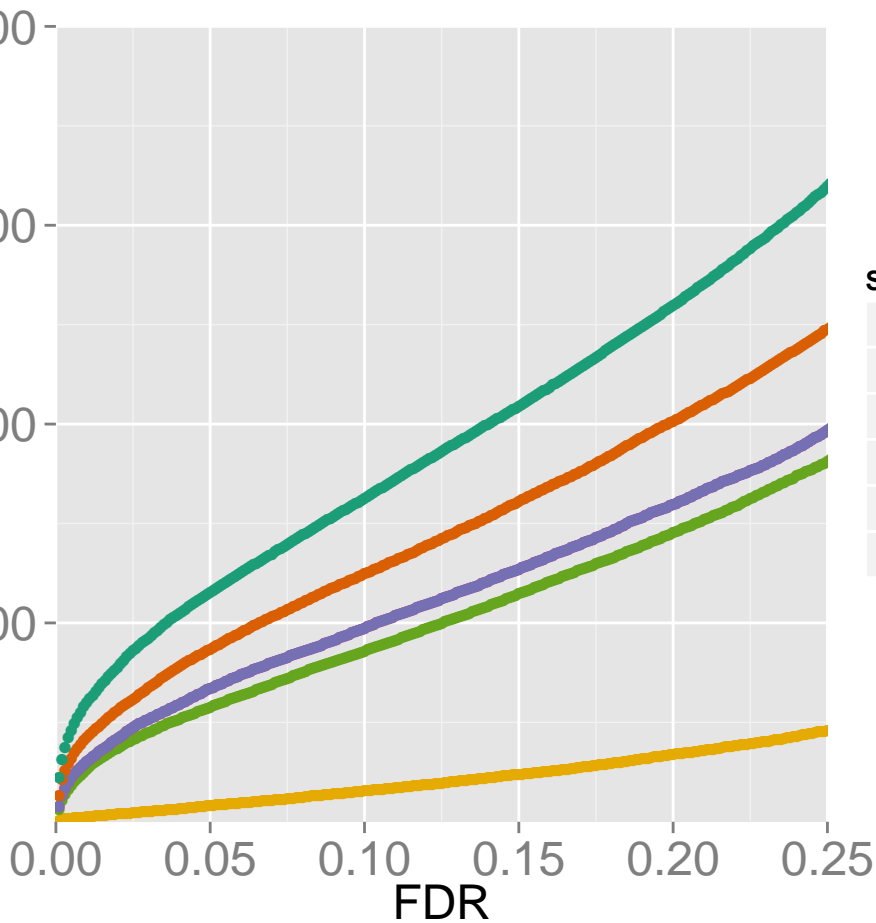

Supplement: Figure S3 — The number of significant QTLs found as a function of false discovery rate (FDR), plotted for the raw data and after each stage of the data normalization procedure that we used (see Methods for details of the method). We first normalised the binding intensities for each sample by the total read depth for that sample. We then corrected for GC composition by removing the median count of binding regions in the same GC bin (100 bins in total) from each binding region. The measures for each binding region were then centre-scaled by removing the mean and then dividing by the standard deviation (track hidden behind GC as center scale does not affect regression). This was followed by a quantile normalization, which maps the measures of each sample to normal quantiles across all binding regions. Lastly, we removed the first principal component that explains the most global phenotypic variation. (PDF) [file pgen.1004798.s003.pdf]

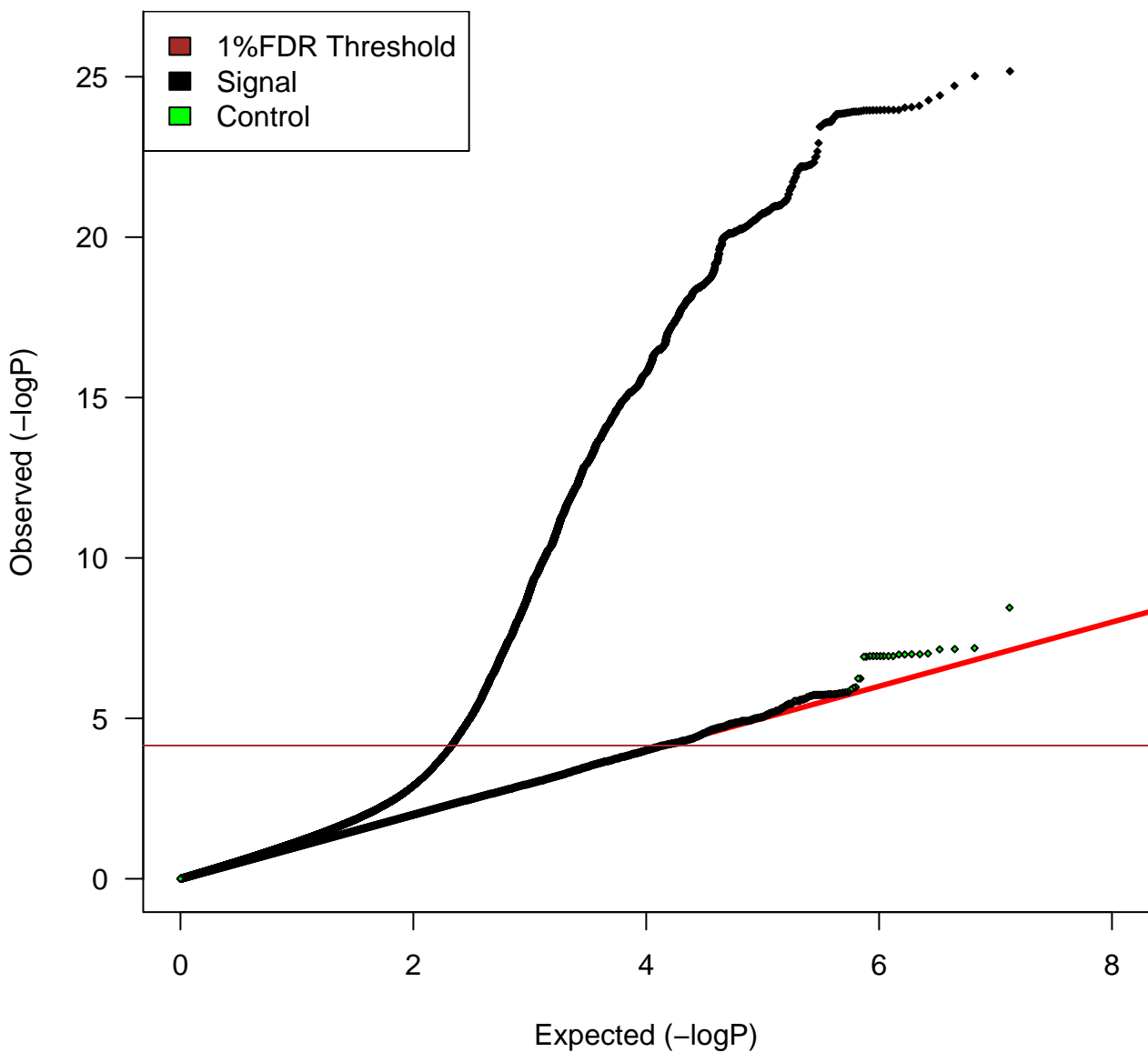

Supplement: Figure S4 — QQ plot for all associations between CTCF binding intensities and genotypes of variants within 50 kb to the centre of binding sites. Purple and green dots indicate P values from actual tests and permutation controls - where sample labels are randomly permuted. We used 1% FDR (brown line) as our cutoff for results. (PDF) [file pgen.1004798.s004.pdf]

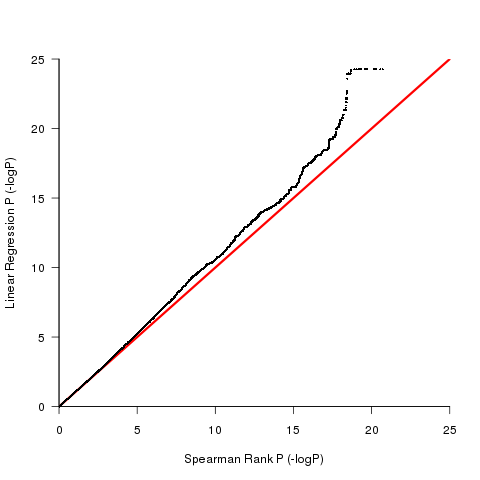

Supplement: Figure S5 — Spearman rank test for association is more conservative but gives similar results. Association test by linear methods can be inappropriate and gives spurious signal if the normality assumption is not met. Although in our normalization procedure the binding measures are mapped to normal quantiles sample-wise, it is still possible that the normality assumption does not hold binding region-wise. To test if this would bias the QTL mapping we performed the same tests using the Spearman rank method. The P values from both sets are sorted and then plotted against each other as Y-axis for the linear test and X-axis for the Spearman rank test. We see a slight elevation of the black line, suggesting the rank test is more conservative but would give similar results, and our linear test is mostly appropriate. (PNG) [file pgen.1004798.s005.png]

$-\log(P)$  distal QTL variant

$-\log(P)$  proximal variant in LD

**D prime**

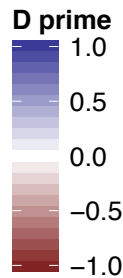

1.0  
0.5  
0.0  
-0.5  
-1.0

**Frq**

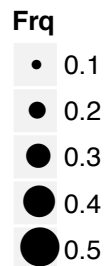

0.1  
0.2  
0.3  
0.4  
0.5

Supplement: Figure S6 — P value distribution of the proximal variants. Here the P values from the association between the CTCF binding and the lead distal QTL variants are plotted against that of the proximal variants, which are in LD with the distal QTL variants. The horizontal and vertical dashed lines are the 1% genome wide FDR threshold established in the main analysis. The diagonal line assists to indicate same P values. Each dot is colored by its D′ value of LD with its size scaled by the allele frequency of the proximal variant. (PDF) [file pgen.1004798.s006.pdf]

cor with motif=0.361

Frequency

10

5

0

-10

-5

0

5

10

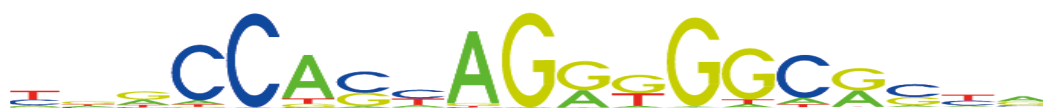

Position

Supplement: Figure S7 — Distribution of the proximal variants that are on motif and in LD with the distal lead QTL variants. Here the proximal variants were aligned to the motif positions. We saw a correlation between their distribution and the information content of the motif at ρ = 0.36. (PDF) [file pgen.1004798.s007.pdf]

A

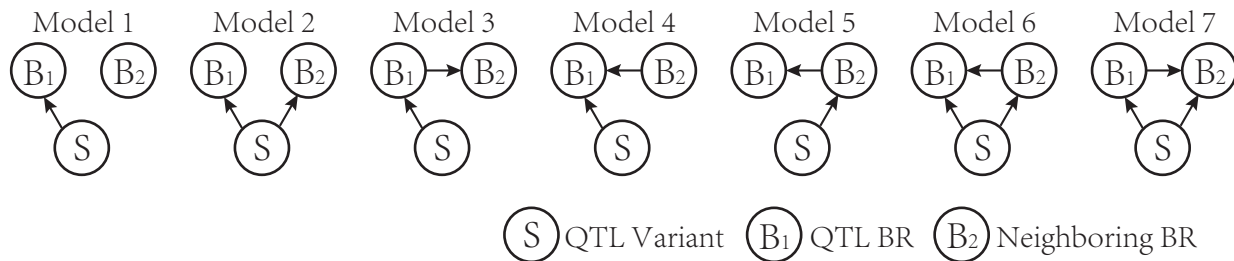

B

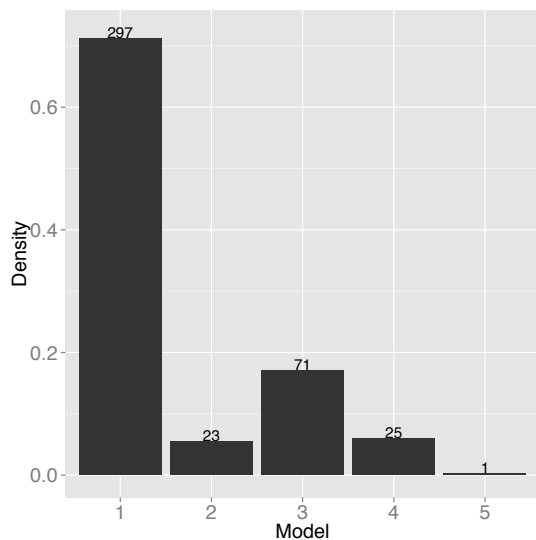

C

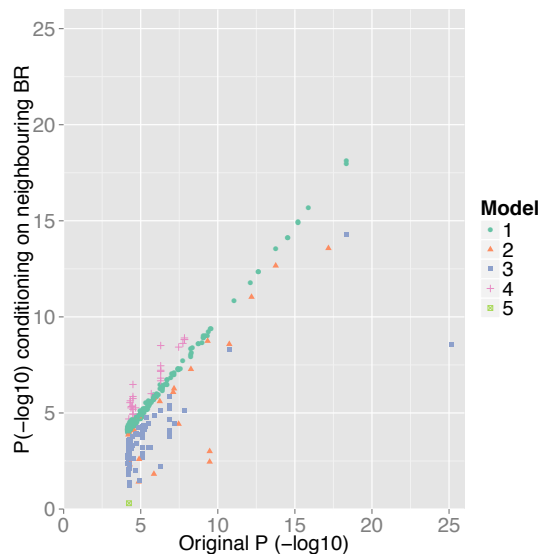

Supplement: Figure S8 — Evidence for indirect effects when a second binding region is present in the distal QTL window. Many (75.5%) of our distal QTLs contain a second CTCF binding region in their 50 kb cis-window. To explore possible causal relationships between the lead variant, the associated binding region(BR1) and the second binding region(BR2) we constructed seven graphical models (A) and compared them using the Bayesian Information Criterion (BIC). In each case we assign the most likely model, chosen as having the lowest BIC. The frequency of the chosen models (B) suggests that there is almost never evidence for the association effect of the distal variant being mediated via a secondary binding region. The most frequently preferred model (1) did not involve BR2 at all; for the next most preferred models (3 and 4) there was some evidence of interactions between neighbouring CTCF binding sites, but we could not explain the variant association to BR1 binding via BR2. The only models which support mediation of binding at BR1 via BR2 are 5 and 6, and in only one case do we see one of these being selected. The P value of BR1 when conditioned on BR2 is plotted in (C). We further investigated the enrichment of a range of ENCODE [1] signals over the QTL binding region and the neighboring region. We found the association between two binding regions (model 3,4) tend to correlate with the active regulatory signals (Figure S9). (PDF) [file pgen.1004798.s008.pdf]

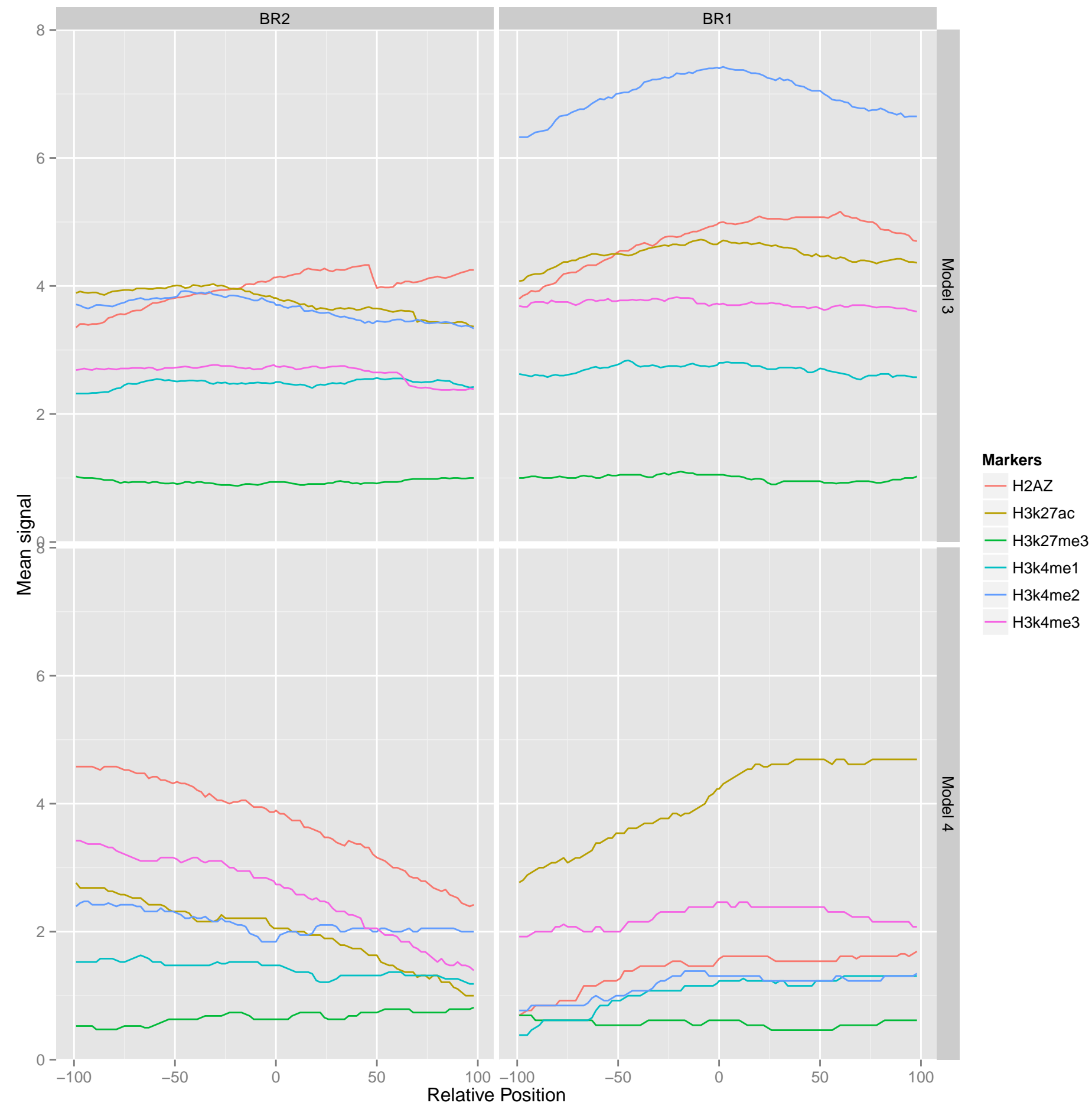

Supplement: Figure S10 — Change of histone modifications depending on the interaction models between the QTL binding region and the neighboring binding region (see Figure S8 and S9 for explanations about the models). (PDF) [file pgen.1004798.s010.pdf]

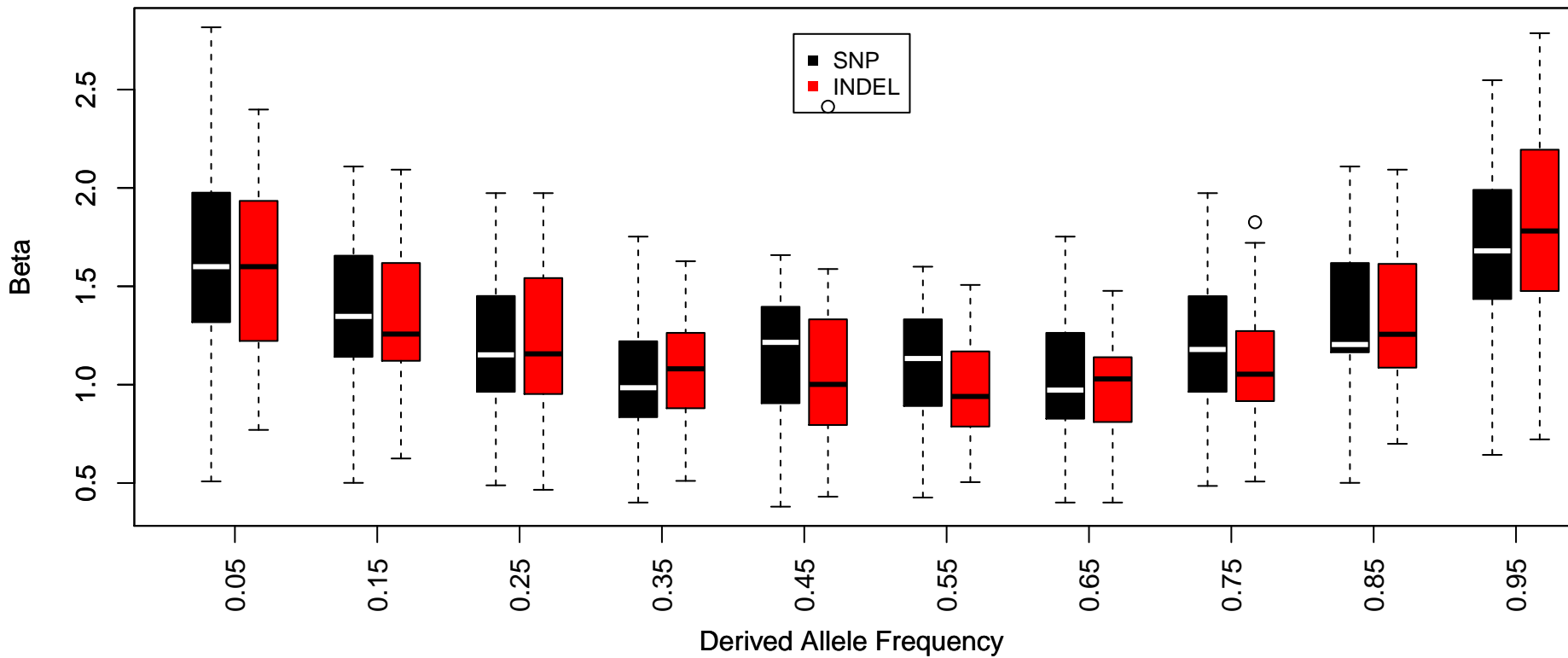

Supplement: Figure S11 — Effect size versus derived allele frequency for all CTCF QTLs identified at 1% FDR. (PDF) [file pgen.1004798.s011.pdf]

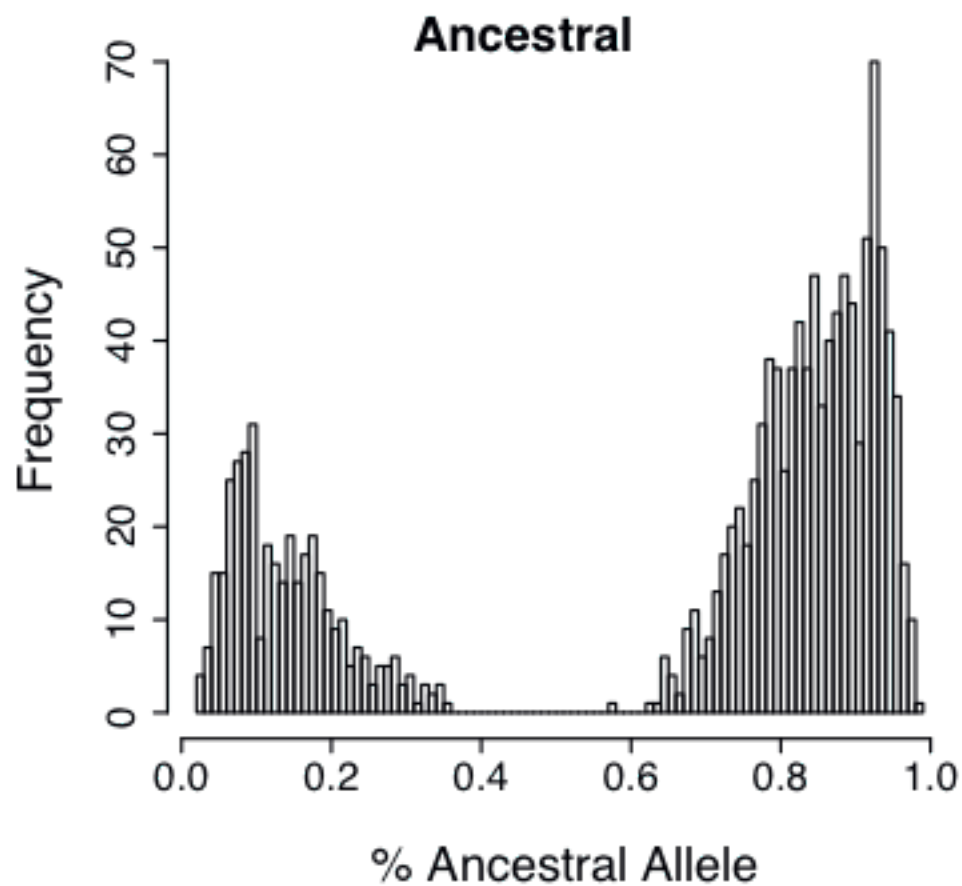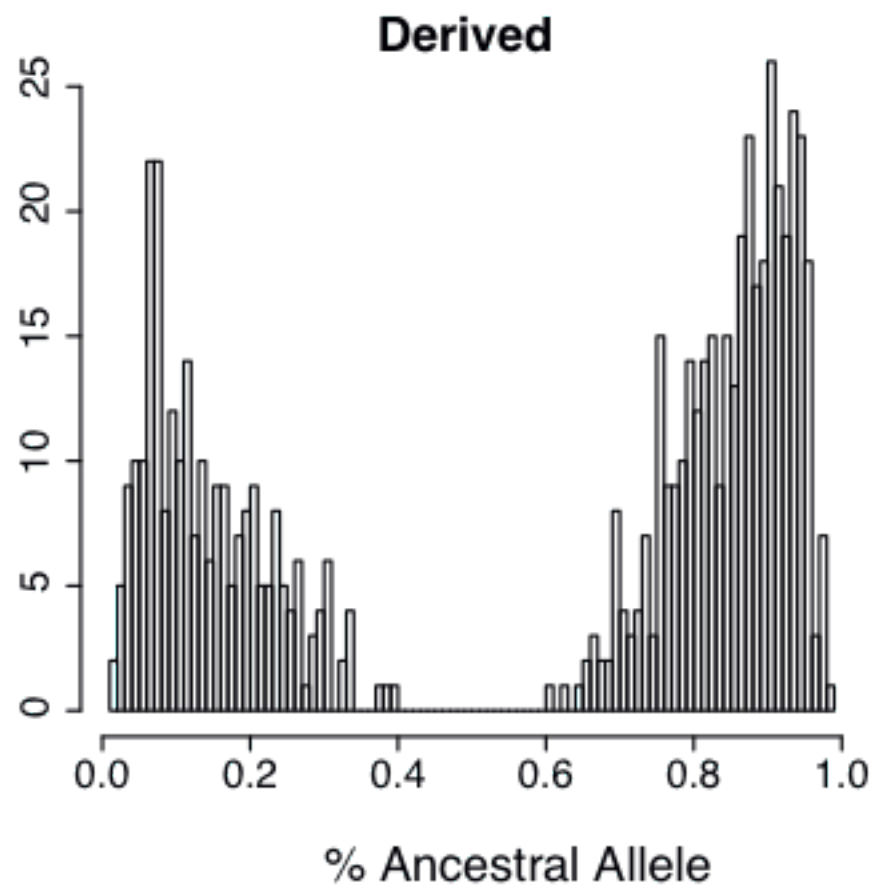

Supplement: Figure S12 — Effect of the Reference Allele. Even when the reference allele is the derived allele (Derived), the binding bias remained towards the ancestral allele. (PDF) [file pgen.1004798.s012.pdf]

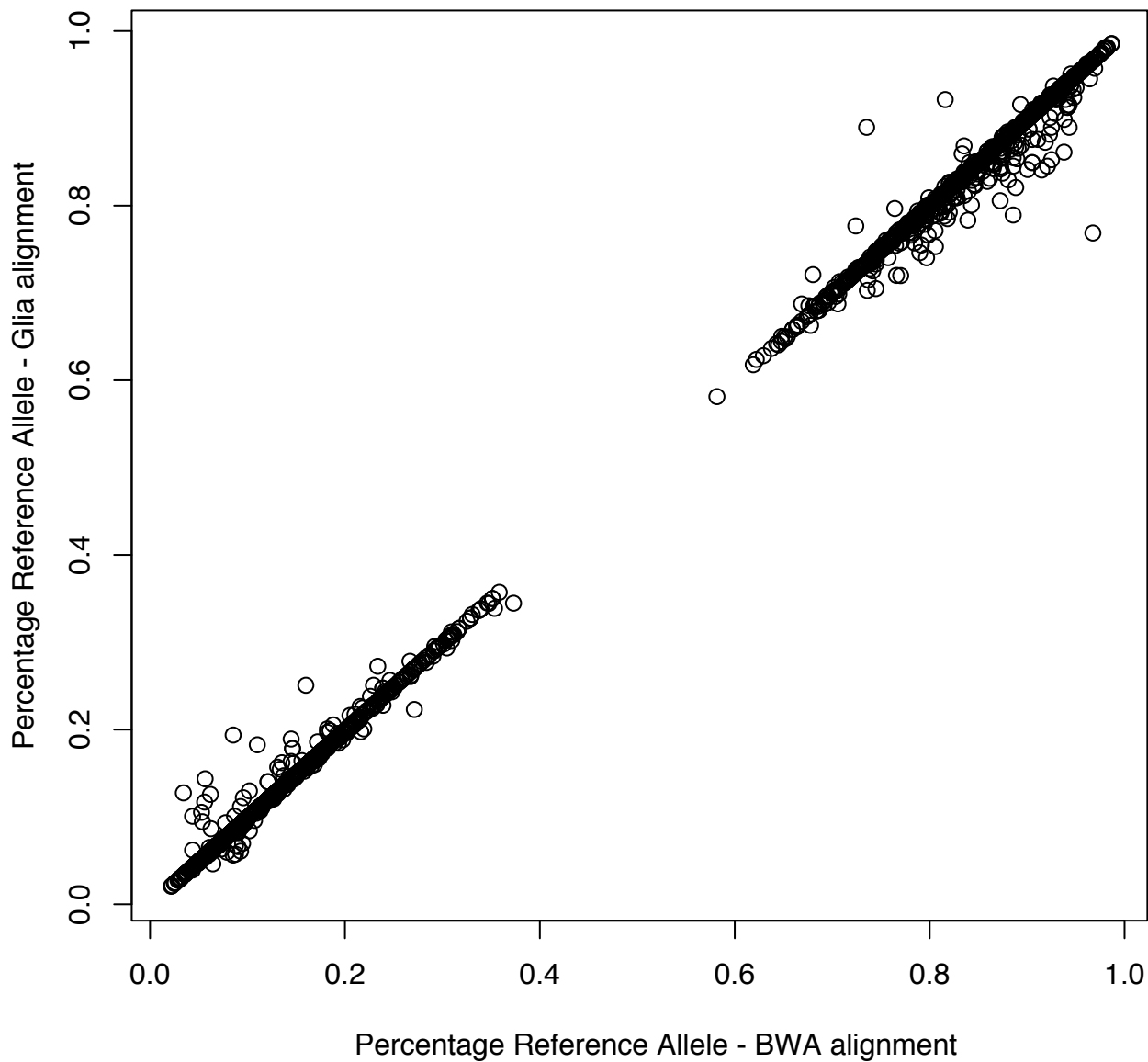

Supplement: Figure S13 — Effect of alignment to allele specific analysis. We performed local realignment using a variant aware aligner glia (https://github.com/ekg/glia) and compared the allelic bias in our significant allele specific sites between the two alignments. We saw that the effect of local realignment is minimum. (PDF) [file pgen.1004798.s013.pdf]

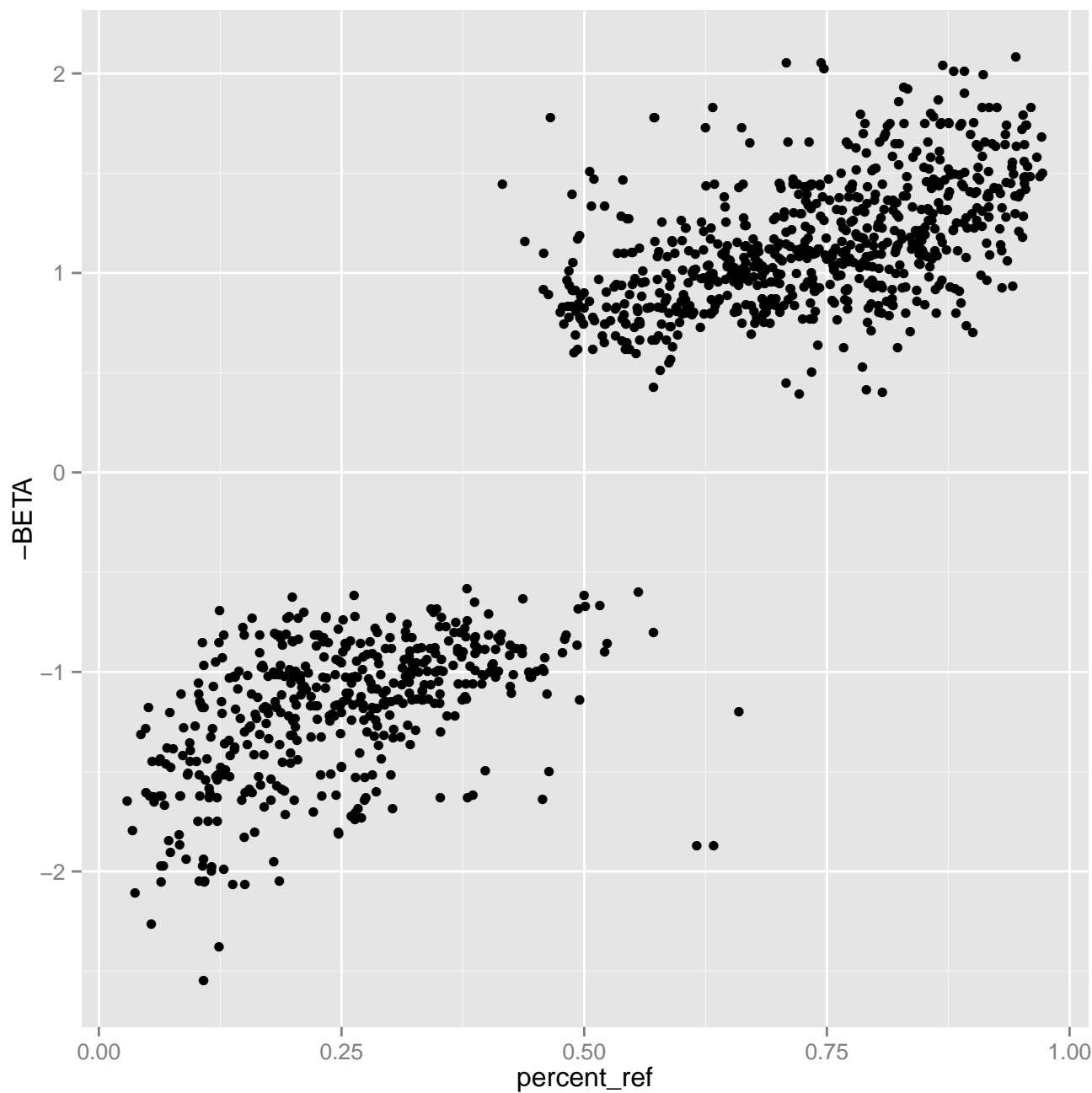

Supplement: Figure S14 — No QTLs with strong effect size in binding regions that do not show strong allele specificity. The x-axis shows allele specificity (measured as % reference), and the y-axis shows between-individual effect (beta) orientated such that positive is towards reference. (PDF) [file pgen.1004798.s014.pdf]

**Merged binding region count vs  $P$ -value**

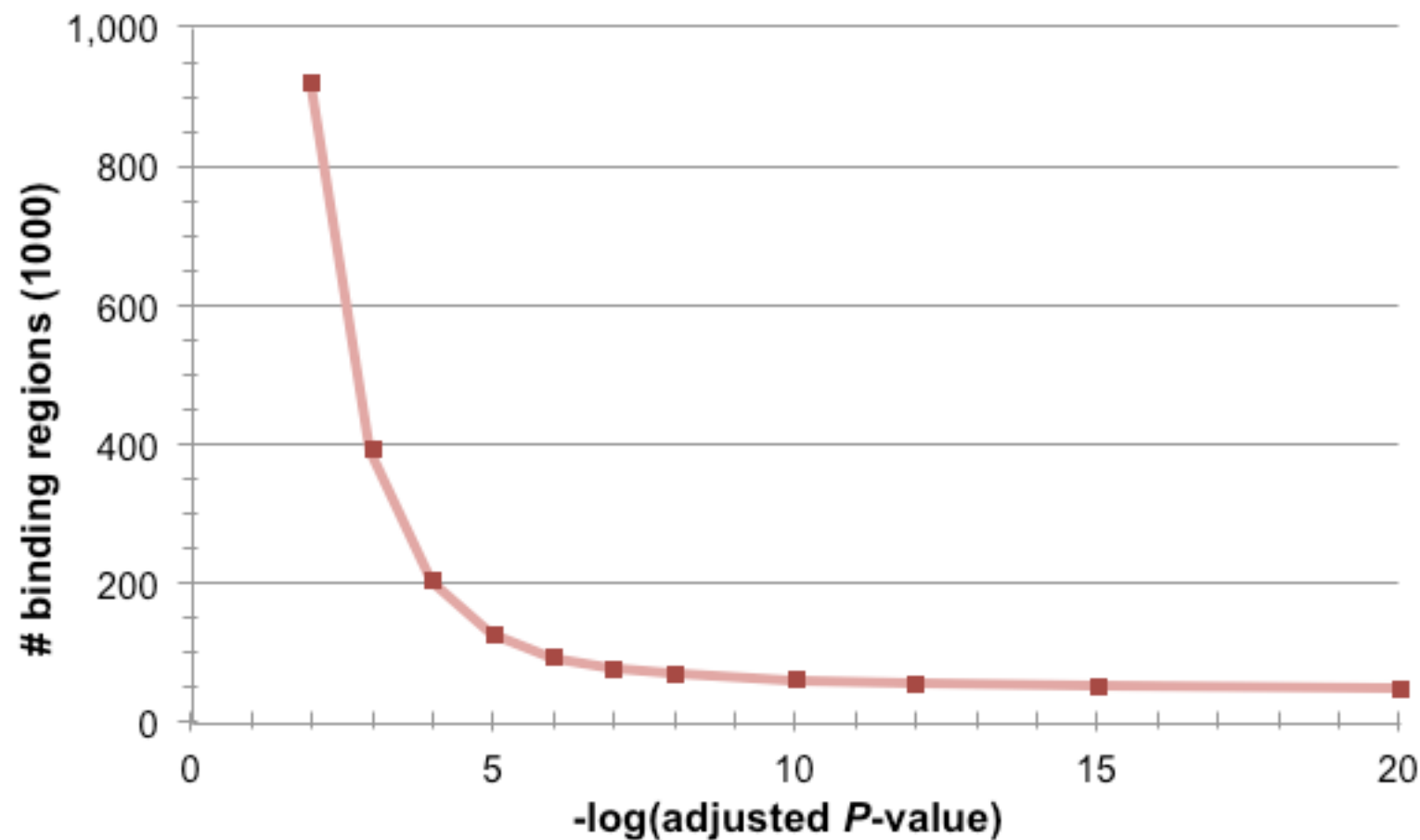

Supplement: Figure S15 — Number of merged binding regions plotted as a function of −log(BH-adjusted binomial P-value). (PDF) [file pgen.1004798.s015.pdf]

## number of contributing cell lines

1 2+ 3+ 4+ 5+ 6+ 7+ 8+ 9+ 10+

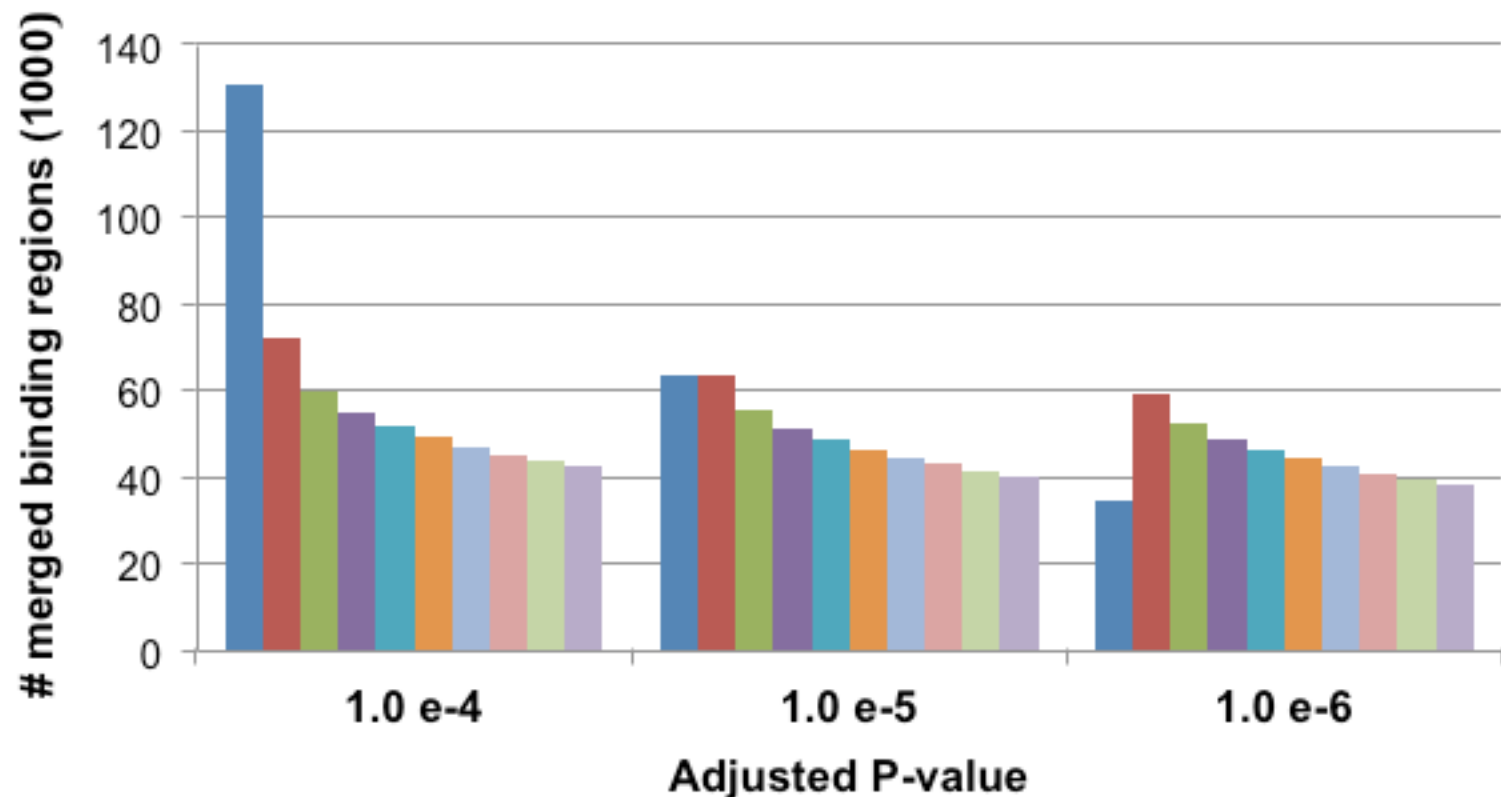

Supplement: Figure S16 — Number of merged binding regions as a function of number of calling cell lines, at three adjusted P-values. (PDF) [file pgen.1004798.s016.pdf]

## number of contributing cell lines

1 2+ 3+ 4+ 5+ 6+ 7+ 8+ 9+ 10+

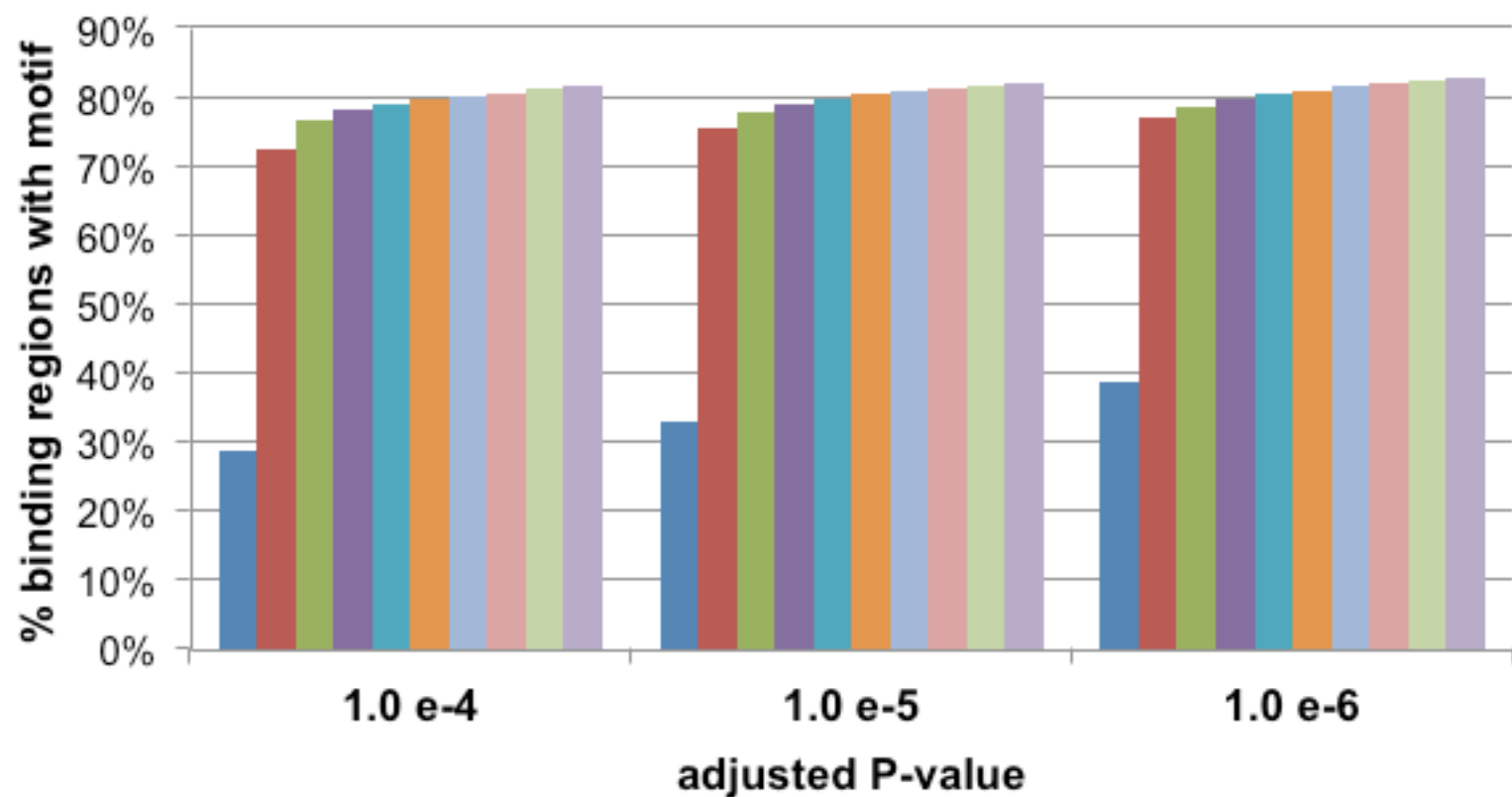

Supplement: Figure S17 — Proportion of merged binding regions as a function of number of calling cell lines, at three adjusted P-values. (PDF) [file pgen.1004798.s017.pdf]

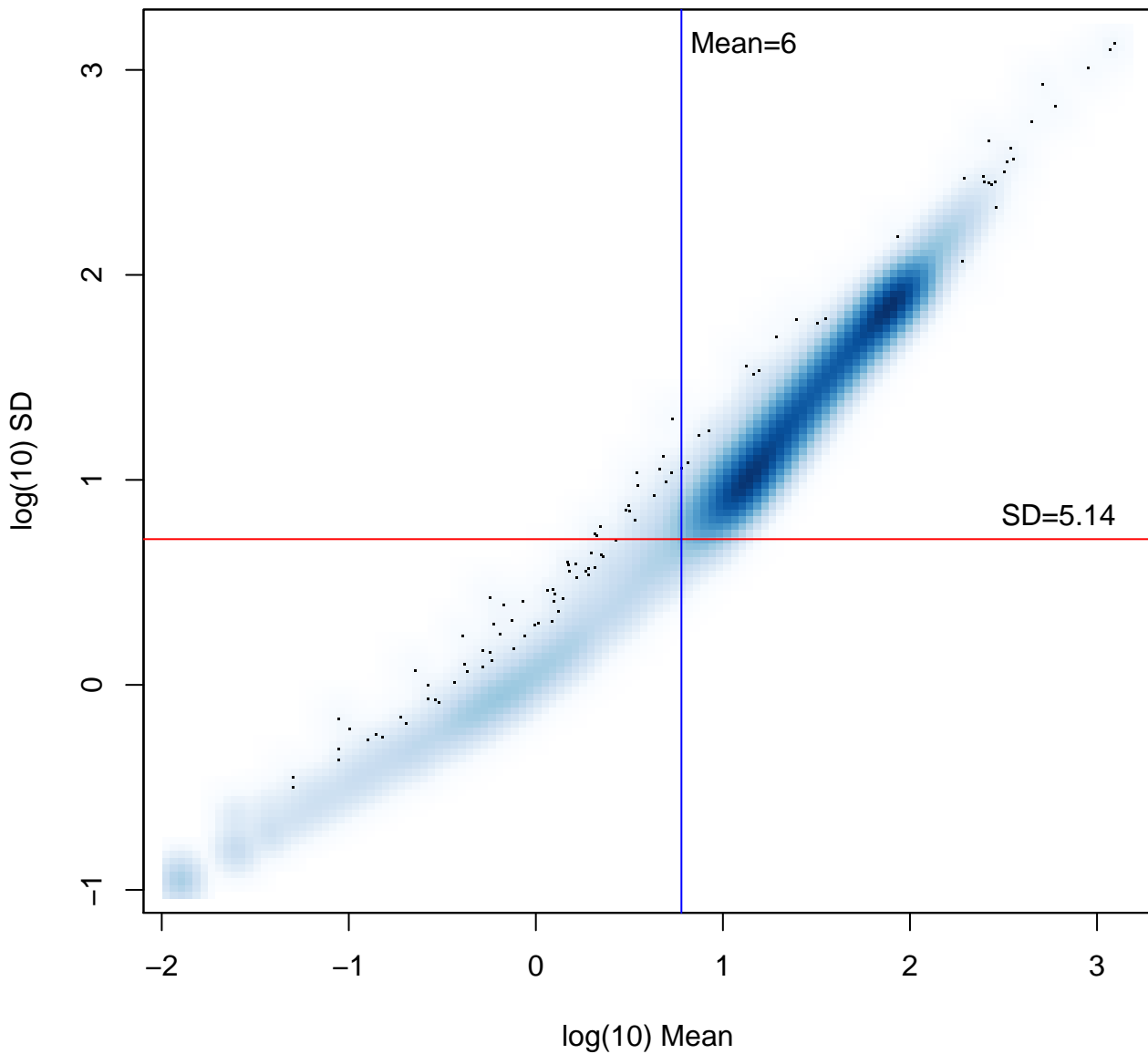

Supplement: Figure S18 — Quality control by raw signal intensity and inter cell line variability. For each binding region we counted the overlapping sequencing fragments (identified by a properly paired read pair) and used it as a measure for the raw binding intensity. We plot the log of the variance of the binding intensities across 51 individuals versus the log of the mean of the binding intensities using the R function smoothScatter. The degree of blue is proportional to the density of data points. As a Poisson process the mean and variance correlate with each other. There exists a natural cut-off between the lower left tail and the majority at mean 6 and standard deviation 5.14. These lower left tail binding regions are the sites with very low intensity and also low variability. We removed these sites, 4,516 binding regions in total, before further analysis. (PDF) [file pgen.1004798.s018.pdf]

# Histone modifications

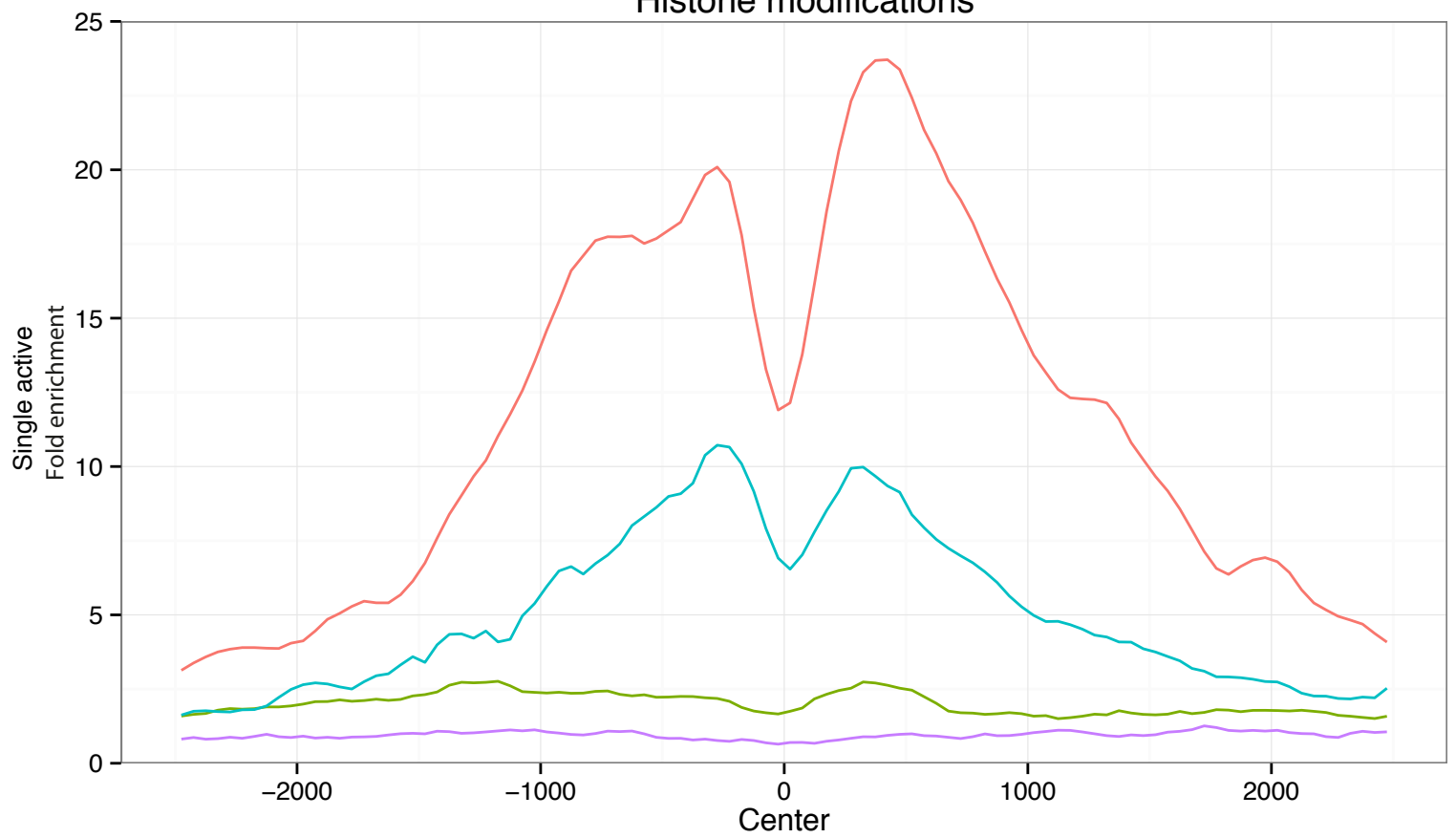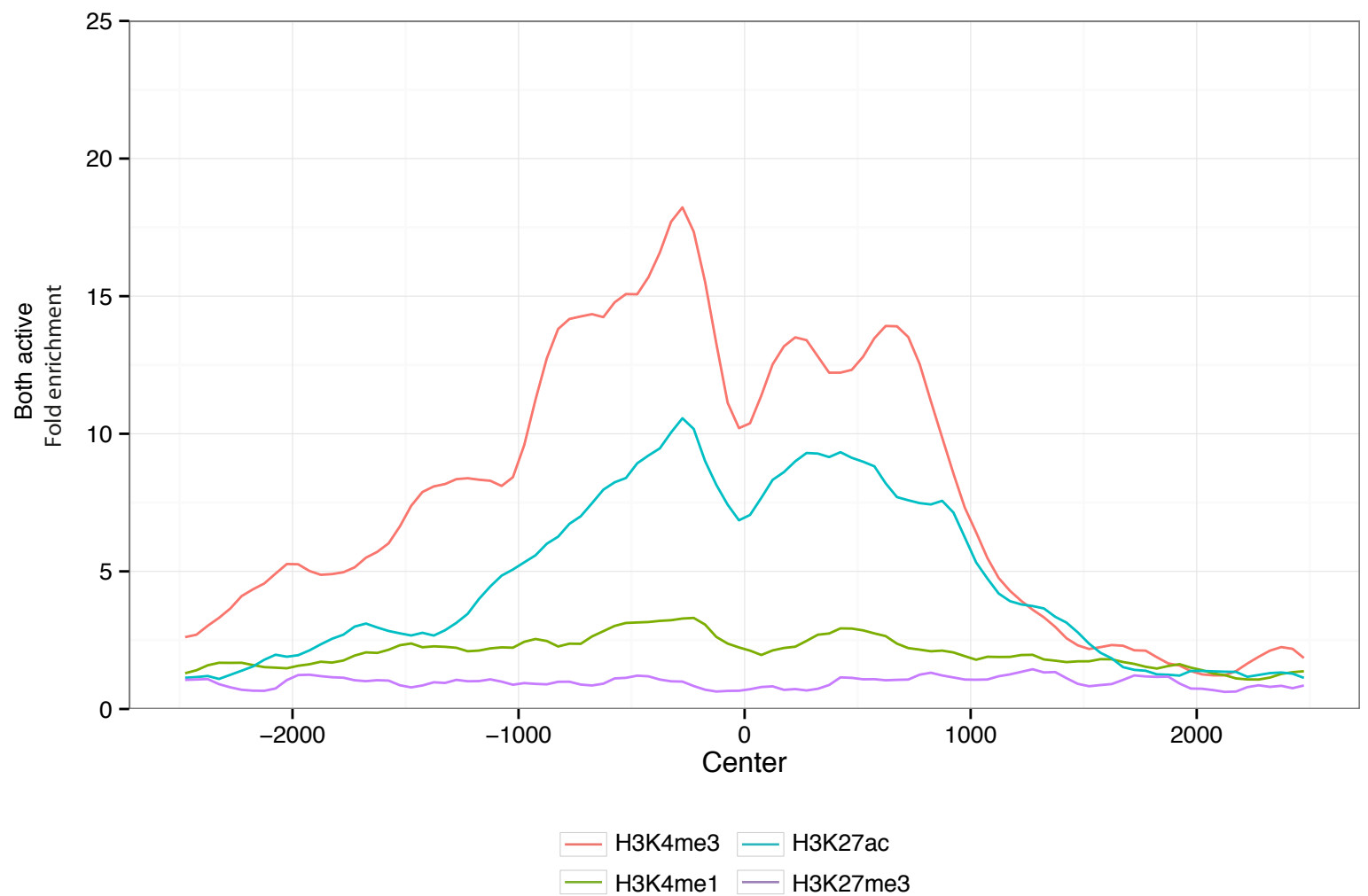

Supplement: Figure S19 — Aggregated signals for histone modifications at X chromosome binding regions split by single-active (top panels) and both-active (bottom panels) CTCF classes for binding regions overlapping promoters. The average ENCODE signal, in GM12878, is determined by the average fold enrichment for this region against random Poisson distribution with local lambda [2]. (PDF) [file pgen.1004798.s019.pdf]

# SMC3

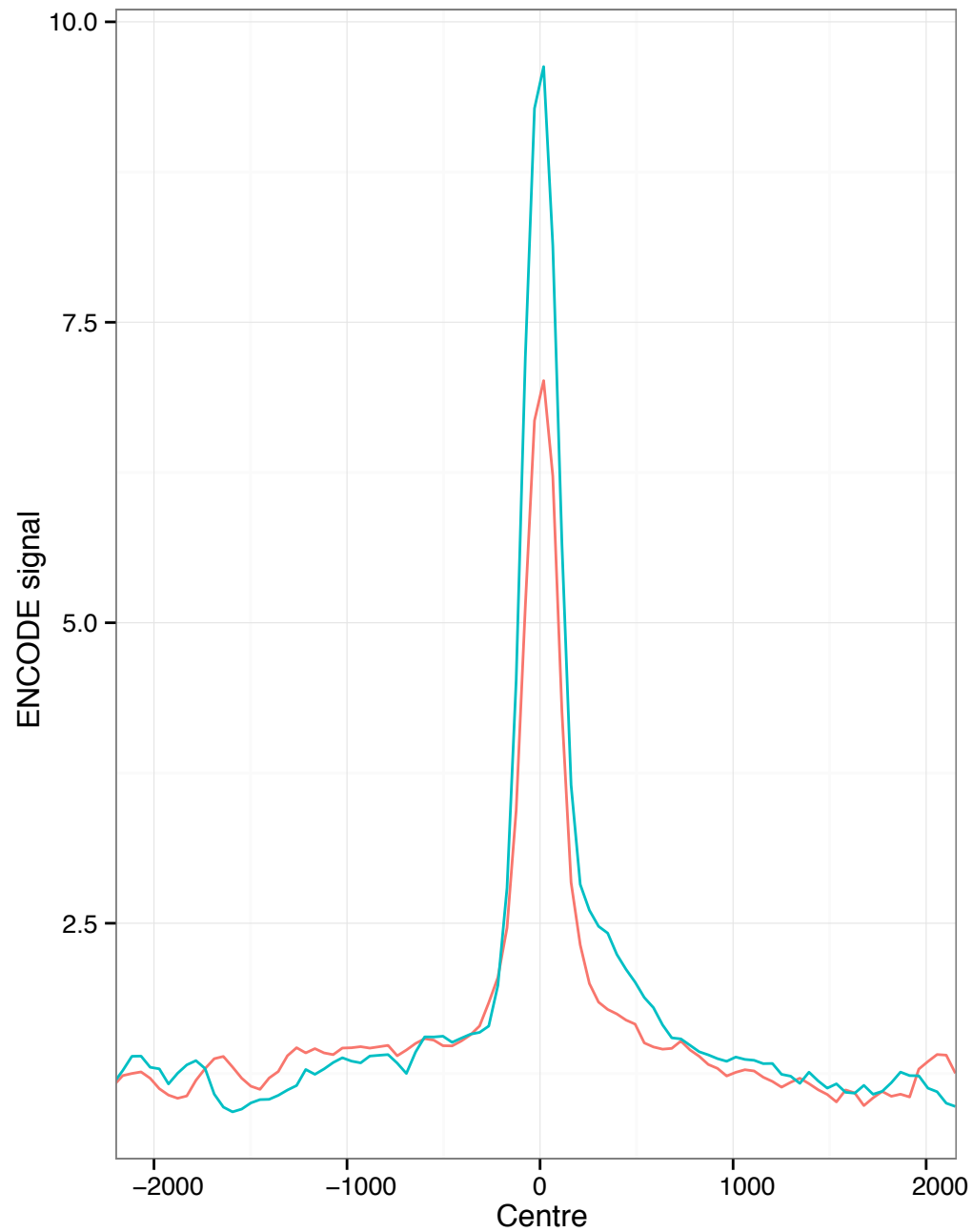

# Rad21

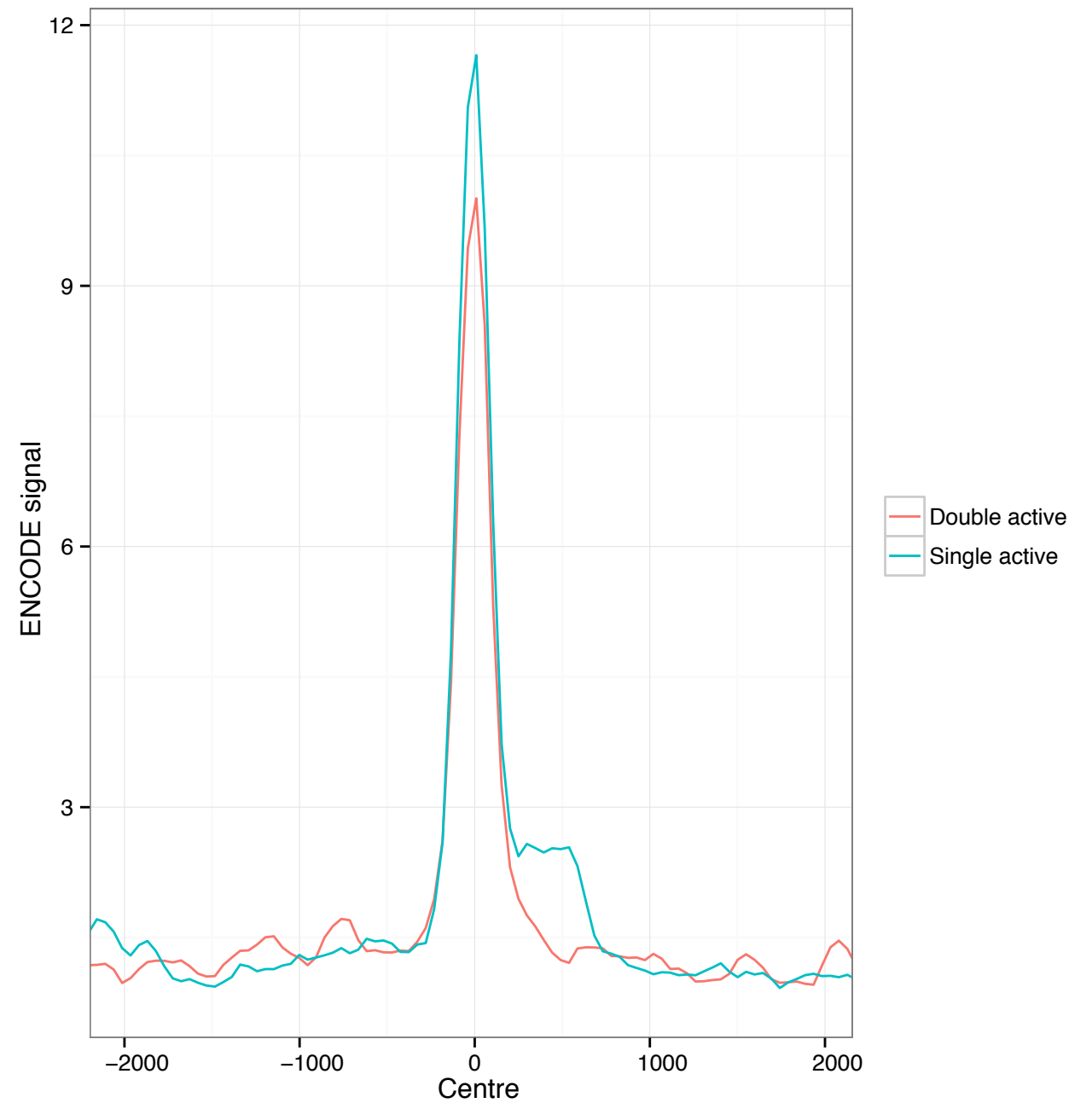

Supplement: Figure S20 — Aggregated signal for transcription factors SMC3 and Rad21 at X chromosome shown for single active and double active binding regions. We plot the aggregated average ENCODE signal in GM12878, which is determined by the average fold enrichment for this region against random Poisson distribution with local lambda [2]. Single active CTCF sites tend to have a small increase in binding. (PDF) [file pgen.1004798.s020.pdf]

# Peak quantifications scaled for library size

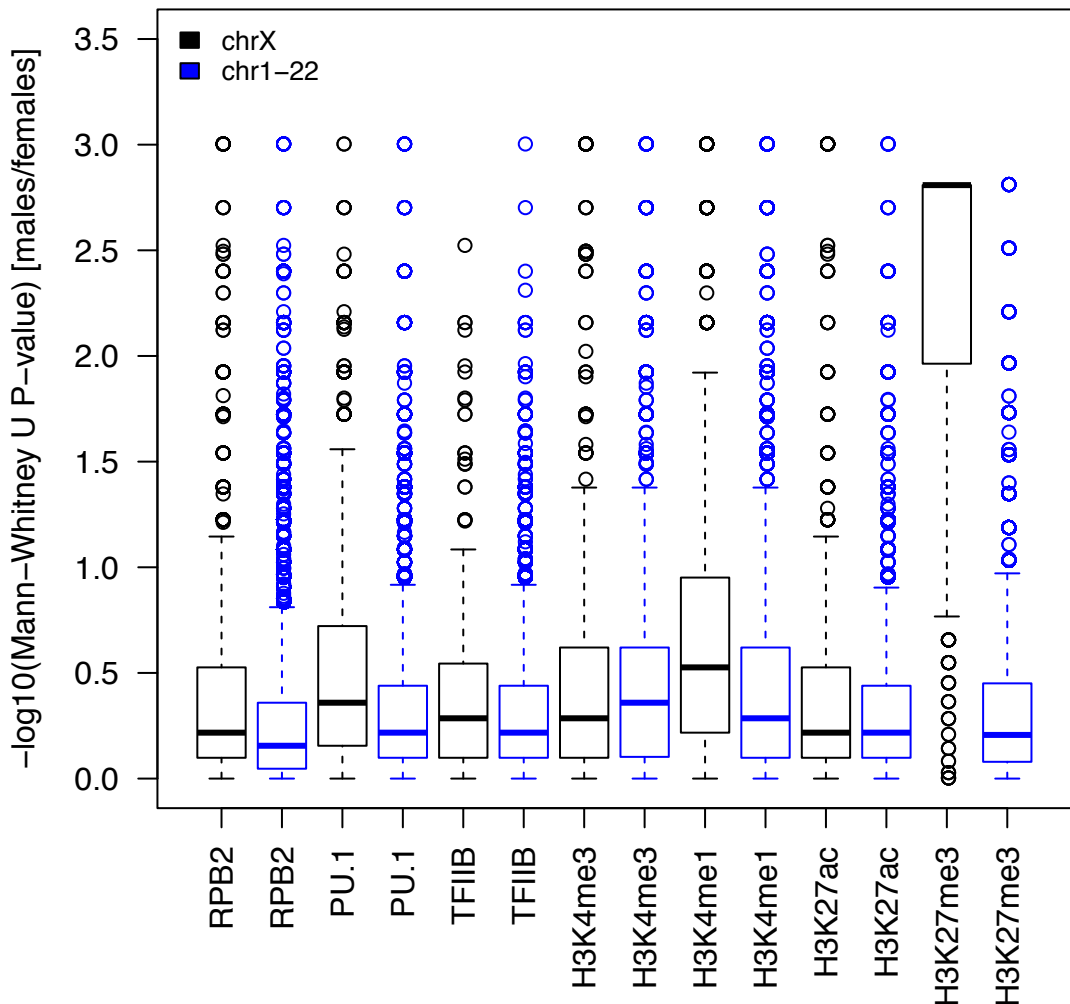

Supplement: Figure S21 — Gender difference in transcription factor binding and histone modification. Using data from [2] (5 males and 5 females, all unrelated), we compared the average signals between males and females on the X (black) and autosome (blue) for a range of markers with data obtained from the ENCODE project [1]. For each marker, all data is used, irrespective of overlapping with CTCF binding. Mann Whitney test is performed separated data on gender. A significant Mann Whitney test indicates a gender specific marker binding. We observed minimum gender specific signals, except for H3K27me3. (PDF) [file pgen.1004798.s021.pdf]

gm12749

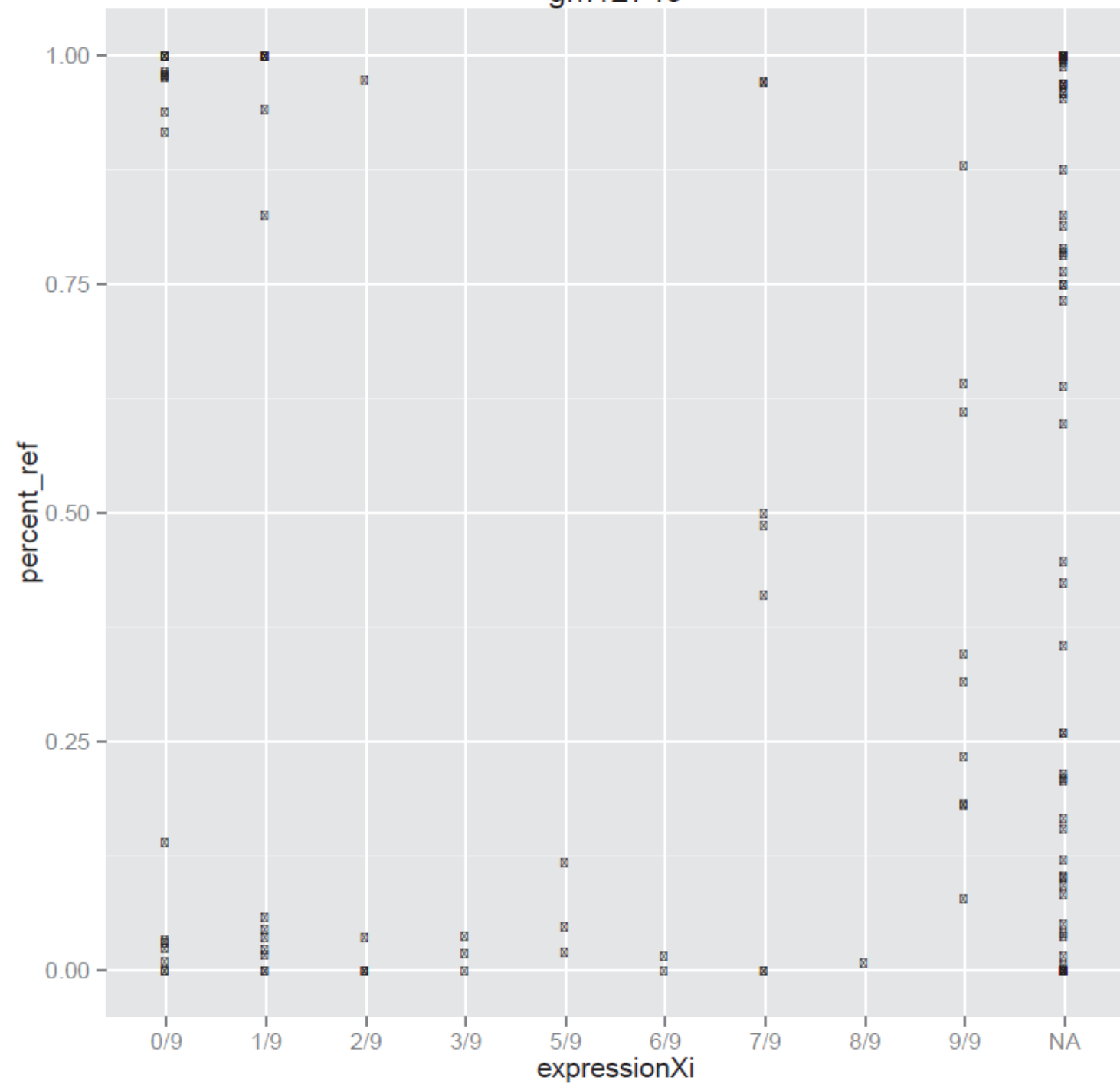

gm12761

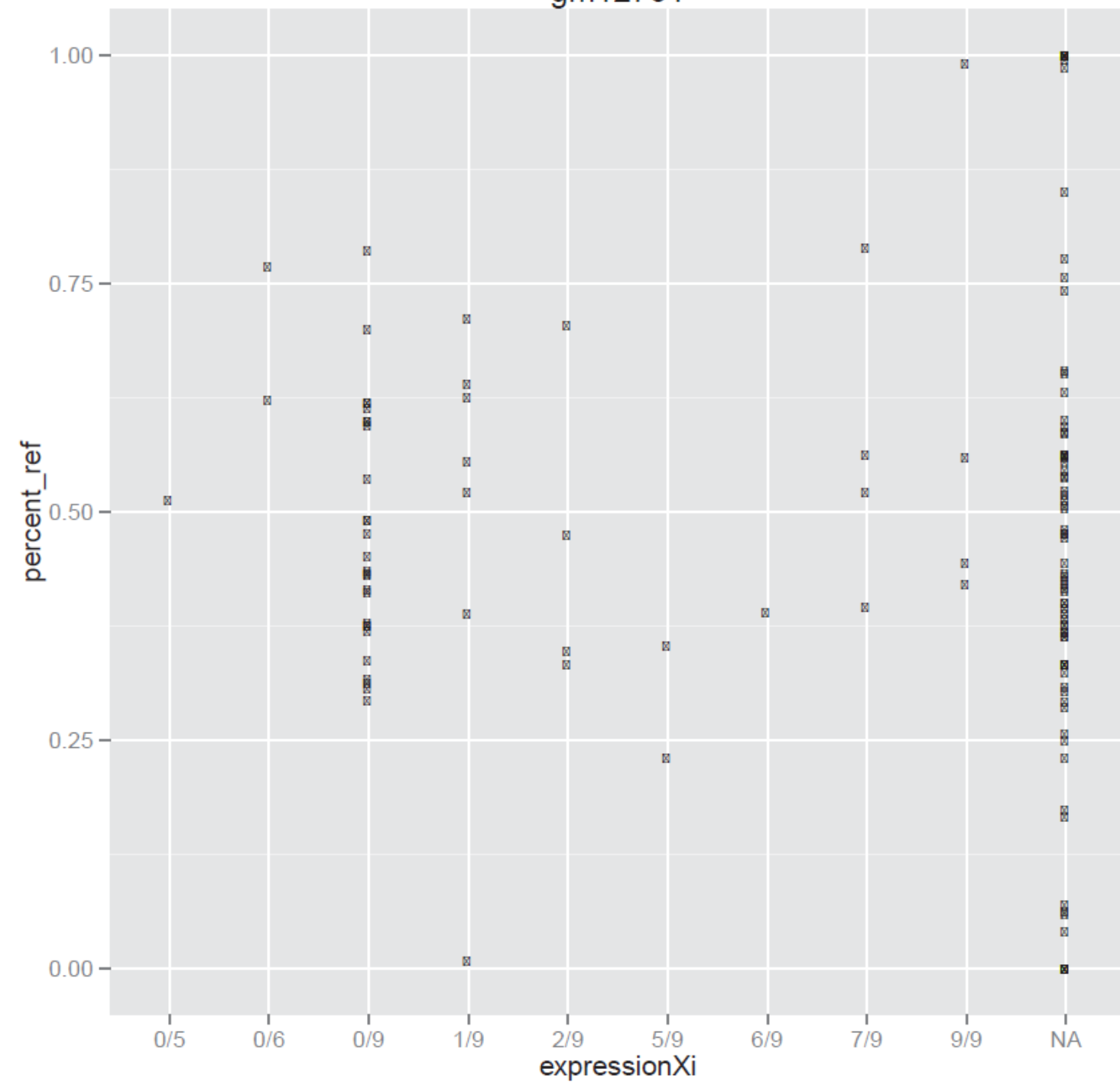

Supplement: Figure S22 — Examples of clonal and polyclonal cell lines. X chromosome genes are grouped according to their expression on the inactivated X [3]. On the x axis, 0/9 are the most strictly X inactivated genes and 9/9 are the genes that show consistent expression from the inactivated X. ‘NA’ denote the genes whose X inactivation status was not determined. On the y axis, percent reference reads from RNA-seq data were counted on the heterozygous SNP sites within those genes and plotted against their X inactivation status. NA12749 on the left was determined to be a clonal sample and NA12761 on the right was determined to be a polyclonal sample. (PDF) [file pgen.1004798.s022.pdf]

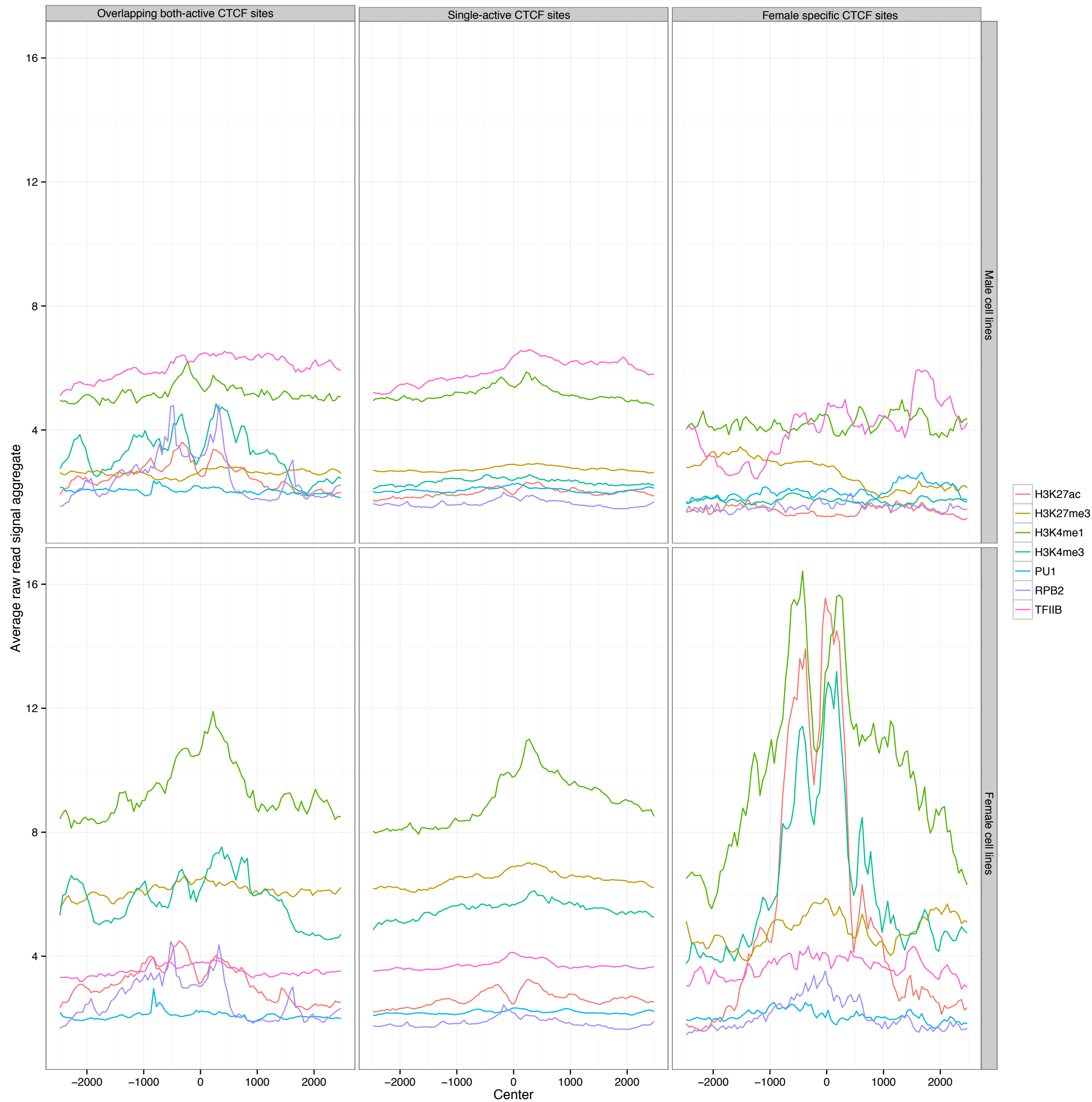

Supplement: Figure S23 — Aggregated signal for transcription factors using data from [2] (5 males and 5 females, all unrelated). We plot the average raw read signal, for several markers in regions that overlap CTCF binding region. Aggregate plots are separated on gender and CTCF classification. We observe that for the both-active and single-active CTCF sites there is, as expected, double as much signal for female than for male cell lines. For regions that show female specific CTCF binding, the aggregated signal track show a change in binding profile for H3K4me3, H3K4me1, and H3K27ac. (PDF) [file pgen.1004798.s023.pdf]
