# Supplementary material for: Quantitative Genetics of CTCF Binding Reveal Local Sequence Effects and Different Modes of X-Chromosome Association
Source: PLoS Genet. 2014 Nov 20;10(11):e1004798. doi: 10.1371/journal.pgen.1004798 (PMC4238955; doi:10.1371/journal.pgen.1004798)
Supplement: Figures S9 — The interaction between QTL binding region and neighboring binding region correlates with regulatory events. The distal QTL set is as previously described (Figure S8). For each of the four categories with sufficient abundance (model 1, 2, 3 and 4), we compare the average signals between the QTL binding region (B1) and the neighboring binding region (B2) for a number of molecular markers using data obtained from the ENCODE project [1]. We observed distinct patterns of regulatory signals between model 1,2 and model 3,4. We saw that when there exists interactions between two binding regions (model 3,4), active transcription factors, enhancers and active histone markers tend to be more enriched in the QTL binding regions, as shown in red. This change is not driven by their distances being closer to the transcription start site (TSS) by chance, measured as the distance to the closest TSS, because the neighboring binding regions have similar distance to the TSS as the QTL binding regions (red and green lines in the density plots). Some of the histone modifications (H2AZ, H3k27ac, H3k4me1, H3k4me2 and H3k4me3) swap enrichment direction between model 3 and model 4 depending on the direction of interaction between B1 and B2 (also see Figure S10 for more detailed enrichment signals). (PDF) [file pgen.1004798.s009.pdf]

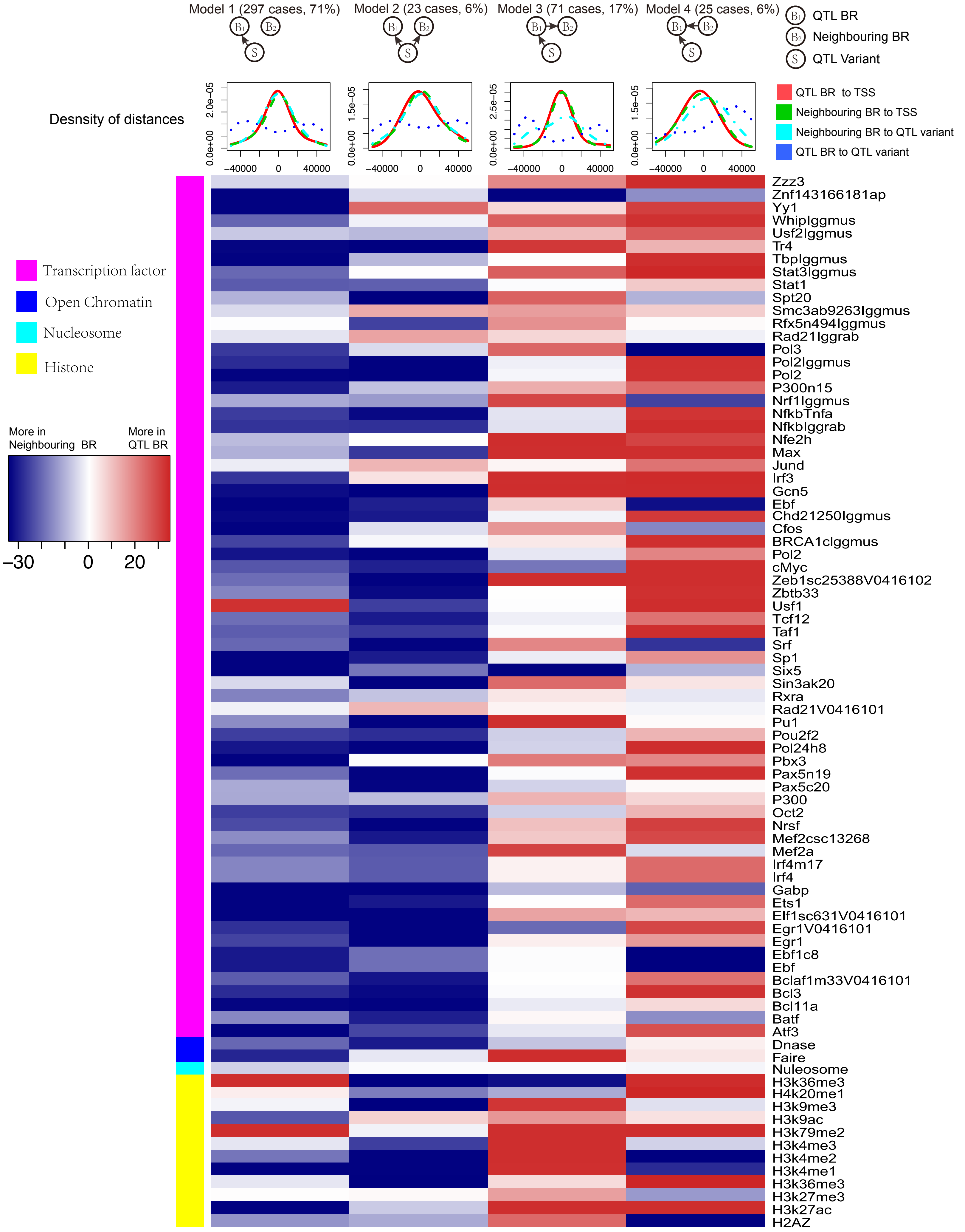
Model 1 (297 cases, 71%)
Model 2 (23 cases, 6%)
Model 3 (71 cases, 17%)
Model 4 (25 cases, 6%)
B1 QTL BR
B2 Neighbouring BR
S QTL Variant
Desnsity of distances
QTL BR to TSS
Neighbouring BR to TSS
Neighbouring BR to QTL variant
QTL BR to QTL variant
Transcription factor
Open Chromatin
Nucleosome
Histone
More in Neighbouring BR
More in QTL BR
−30
0
20
Zzz3
Znf143166181ap
Yy1
WhipIggmus
Usf2Iggmus
Tr4
TbpIggmus
Stat3Iggmus
Stat1
Spt20
Smc3ab9263Iggmus
Rfx5n494Iggmus
Rad21Iggrab
Pol3
Pol2Iggmus
Pol2
P300n15
Nrf1Iggmus
NfkbTnfa
NfkbIggrab
Nfe2h
Max
Jund
Irf3
Gcn5
Ebf
Chd21250Iggmus
Cfos
BRCA1cIggmus
Pol2
cMyc
Zeb1sc25388V0416102
Zbtb33
Usf1
Tcf12
Taf1
Srf
Sp1
Six5
Sin3ak20
Rxra
Rad21V0416101
Pu1
Pou2f2
Pol24h8
Pbx3
Pax5n19
Pax5c20
P300
Oct2
Nrsf
Mef2csc13268
Mef2a
Irf4m17
Irf4
Gabp
Ets1
Elf1sc631V0416101
Egr1V0416101
Egr1
Ebf1c8
Ebf
Bclaf1m33V0416101
Bcl3
Bcl11a
Batf
Atf3
Dnase
Faire
Nuleosome
H3k36me3
H4k20me1
H3k9me3
H3k9ac
H3k79me2
H3k4me3
H3k4me2
H3k4me1
H3k36me3
H3k27me3
H3k27ac
H2AZ
